# Supplementary material for: 3-Benzoylisoxazolines by 1,3-Dipolar Cycloaddition: Chloramine-T-Catalyzed Condensation of α-Nitroketones with Dipolarophiles
Source: Molecules. 2021 Jun 8;26(12):3491. doi: 10.3390/molecules26123491 (PMC8227129; doi:10.3390/molecules26123491)
Supplement: Supplementary file 1 [file molecules-26-03491-s001.zip › molecules-1149245-supplementary.pdf]

Supporting Information

for

3-Benzoylisoxazolines by 1,3-Dipolar Cycloaddition: Chloramine -T -Catalyzed Condensation of α-Nitroketones with Dipolarophiles.

Xinhui Pan [1, 2,†], Xiaobing Xin [2,3,†], Ying Mao [4,†], Xin Li [2], Yanan Zhao [2],Yidi Liu [2], Ke Zhang [2] Xiaoda Yang and Jinhui Wang [2*]

[1] Stake Key Laboratory of Natural and Biomimetic Drugs, and Department of Chemical Biology at School of Pharmaceutical Sciences, Peking University, Beijing, 100191, P. R. China; panxhshzu@shzu.edu.cn(X. P.); xyang@bjmu.edu.cn(X. Y.)

[2] Key Laboratory of Xinjiang Phytomedicine Resource and Utilisation, Ministry of Education, School of Pharmaceutical Sciences, Shihezi University, Shihezi 832002, P. R. China; 18699300391@163.com(X. X.); lixin@stu.shzu.edu.cn(X. L.); zynhyl@stu.shzu.edu.cn(Y. Z.); lyd@stu.shzu.edu.cn(Y. L.); xjzk1984@163.com(K. Z.); wangjinhui@hrbmu.edu.cn (J. W.)

[3] Department of Pharmacology, Houbo College of Xinjiang Medical University, Kelemayi, 834000, P. R. China

[4] School of Chemistry and Chemical Engineering, Shandong University, Jinan 250100, P. R. China; maoying1984.@163.com

* Correspondence: wangjinhui@hrbmu.edu.cn

† These authors contributed equally

Table of Contents

1. General Experimental Methods……………………………………………...……S2

2.General procedure for the cycloaddition of alkenes and α-Nitroketones…..…S2

3. Analytical data for Products…………………………………………………..……S2

4. ^{1}H and ^{13}C NMR spectra……………………………………………………… S10

EXPERIMENTAL SECTION

General Experimental Methods. ^{1}H NMR (400 MHz) and ^{13}C NMR (101 MHz) were recorded at room temperature on DRX-400 spectrometer (Bruker, Germany) in $CDCl_3$. The chemical shifts are given in parts per million (ppm) on the delta (δ) scale. The solvent peak was used as a reference value, for ^{1}H NMR: $CDCl_3$ δ 7.26; for ^{13}C NMR: $CDCl_3$ at 77.16 ppm. IR spectra were recorded using an Avatar 360 FT-IR ESP spectrometer (Nicolet, USA) at room temperature. HR-ESI-MS spectra were acquired using an Agilent 6210 ESI/TOF mass spectrometer (Agilent Technologies, Santa Clara, CA, USA). Analytical TLC was run on silica gel plates (GF254, Yantai Institute of Chemical Technology, Yantai, China). Spots on the plates were observed under UV light. Column chromatography was performed on silica gel (200~300 mesh and 300~400 mesh; Qingdao Marine Chemical Factory, Qingdao, China). Super-dry solvent CH_3CN, DMSO and DMF were purchased from Aldrich and used as supplied. The α-nitroketones were synthesized using the same method as reported in the literature[1].

General procedure for the cycloaddition of alkenes and α-Nitroketones.

Chloramine-T (0.0625 mmol, 0.5 equiv) was added to a solution of **1** (0.125 mmol, 1 equiv) and **2** (0.625 mmol, 5 equiv) (or **4** [0.625 mmol, 5 equiv] or **6** [0.625 mmol, 5 equiv]) in CH_3CN (0.2 mL). The mixture was then stirred at 80 ℃ until the starting material disappeared as monitored by TLC. Subsequently, the mixture was directly purified by flash chromatography (with ethyl acetate/petroleum ether as the eluent) to obtain the desired product (3, 5 or 7).

Analytical data for Products

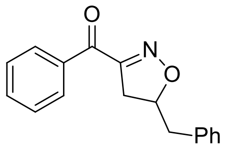

(5-Benzyl-4,5-dihydroisoxazol-3-yl)(phenyl)methanone (3a)

The compound was prepared following the general procedure. Yield 77%. ^{1}H NMR (400 MHz, $CDCl_3$) δ 8.22 – 8.12 (m, 2H), 7.63 – 7.57 (m, 1H), 7.51 – 7.44 (m, 2H), 7.38 – 7.33 (m, 2H), 7.28 (m, 3H), 5.08 (ddt, *J* = 10.8, 7.9, 6.3 Hz, 1H), 3.37 (dd, *J* = 17.6, 10.8 Hz, 1H), 3.19 – 3.08 (m, 2H), 2.98 (dd, *J* = 14.0, 6.5 Hz, 1H); ^{13}C NMR (101 MHz, $CDCl_3$) δ 186.2, 157.5, 135.8, 135.6, 133.3, 130.1(2C), 129.3(2C), 128.5(2C), 128.1(2C), 126.8, 83.3, 40.7, 38.1; IR νmax 3033, 1654, 1581, 710, 672 cm^{-1}; HRMS (EI) *m/z* calcd for $C_{17}H_{16}NO_2$ $[M + H]^+$ 266.1176, found 266.1178. These data are consistent with reported literature values[2].

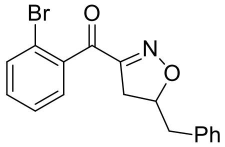

(5-Benzyl-4,5-dihydroisoxazol-3-yl)(2-bromophenyl)methanone (3b)

The compound was prepared following the general procedure. Yield 73%. ^{1}H NMR (400 MHz, $CDCl_3$) δ 7.51 (d, *J* = 7.6 Hz, 1H), 7.29 – 7.25 (m, 2H), 7.24 (m, 2H), 7.21 (m, 1H), 7.18 (m, 2H), 7.15 (m, 1H), 5.05 (ddt, *J* = 11.0, 7.6, 6.3 Hz, 1H), 3.22 (dd, *J* = 17.5, 10.9 Hz, 1H), 3.04 – 2.94 (m, 2H), 2.88 (dd, *J* = 14.0, 6.5 Hz, 1H); ^{13}C NMR (101 MHz, $CDCl_3$) δ 189.2, 158.1, 139.2, 135.8, 133.4, 132.1, 129.8, 129.7(2C), 128.8(2C), 127.2, 127.1, 120.0, 85.2, 41.0, 36.7; IR νmax 3034, 1680, 1585, 755, 694 cm^{-1}; HRMS (EI) *m/z* calcd for $C_{17}H_{15}NO_2Br$ $[M + H]^+$ 344.0281, found 344.0279.

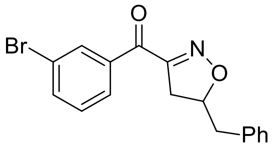

(5-Benzyl-4,5-dihydroisoxazol-3-yl)(3-bromophenyl)methanone (3c)

The compound was prepared following the general procedure. Yield 67%. ^{1}H NMR (400 MHz, $CDCl_3$) δ 8.24 (m, 1H), 8.07 (m, 1H), 7.70 (m, 1H), 7.37 – 7.33 (m, 2H), 7.32 (m, 1H), 7.27 (m, 3H), 5.16 – 5.03 (m, 1H), 3.34 (dd, *J* = 17.6, 10.9 Hz, 1H), 3.16 – 3.05 (m, 2H), 2.98 (dd, *J* = 14.0, 6.4 Hz, 1H); ^{13}C NMR (101 MHz, $CDCl_3$) δ 185.0, 157.6, 137.5, 136.4, 135.9, 133.2, 129.9, 129.6(2C), 128.9,

128.8(2C), 127.2, 122.6, 83.9, 40.9, 38.1; IR νmax 3038, 1691, 1616, 910, 811, 742 cm^{-1}; HRMS (EI) *m/z* calcd for $C_{17}H_{15}NO_2Br$ $[M + H]^+$ 344.0281, found 344.0293.

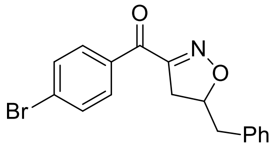

(5-Benzyl-4,5-dihydroisoxazol-3-yl)(4-bromophenyl)methanone (3d)

The compound was prepared following the general procedure. Yield 72%.^{1}H NMR (400 MHz, $CDCl_3$) δ 8.04 – 7.97 (m, 2H), 7.62 – 7.56 (m, 2H), 7.35 – 7.30 (m, 2H), 7.25 (m, 3H), 5.07 (ddt, *J* = 10.9, 7.9, 6.3 Hz, 1H), 3.34 (dd, *J* = 17.6, 10.9 Hz, 1H), 3.13 – 3.05 (m, 2H), 2.96 (dd, *J* = 14.0, 6.5 Hz, 1H).; ^{13}C NMR (101 MHz, $CDCl_3$) δ 185.4, 157.9, 136.1, 134.6, 132.0(2C), 131.9(2C), 129.7(2C), 129.2, 128.9(2C), 127.2, 83.9, 41.1, 38.3; IR νmax 3046, 1696, 1615, 801, 750, 686 cm^{-1}; HRMS (EI) *m/z* calcd for $C_{17}H_{15}NO_2Br$ $[M + H]^+$ 344.0281, found 344.0276.

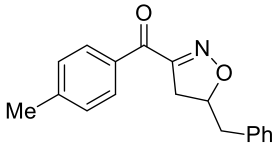

(5-Benzyl-4,5-dihydroisoxazol-3-yl)(p-tolyl)methanone (3e)

The compound was prepared following the general procedure. Yield 68%. ^{1}H NMR (400 MHz, $CDCl_3$) δ 8.08 – 8.03 (m, 2H), 7.33 (m, 2H), 7.28 (m, 2H), 7.25 (m, 3H), 5.05 (ddt, *J* = 10.8, 7.9, 6.3 Hz, 1H), 3.35 (dd, *J* = 17.6, 10.8 Hz, 1H), 3.15 – 3.07 (m, 2H), 2.96 (dd, *J* = 14.0, 6.6 Hz, 1H), 2.42 (s, 3H); ^{13}C NMR (101 MHz, $CDCl_3$) δ 186.1, 157.9, 144.7, 136.2, 133.4, 130.6(2C), 129.6(2C), 129.2(2C), 128.8(2C), 127.1, 83.5, 41.1, 38.6, 21.9; IR νmax 3023, 2923, 1650, 1600, 831, 750, 693 cm^{-1}; HRMS (EI) *m/z* calcd for $C_{18}H_{18}NO_2$ $[M + H]^+$ 280.1332, found 280.1336.

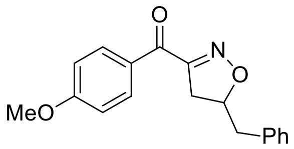

(5-Benzyl-4,5-dihydroisoxazol-3-yl)(4-methoxyphenyl)methanone (3f)

The compound was prepared following the general procedure. Yield 54%. ^{1}H NMR (400 MHz, $CDCl_3$) δ 8.23 – 8.15 (m, 2H), 7.36 – 7.30 (m, 2H), 7.29 – 7.25 (m, 3H), 6.97 – 6.91 (m, 2H), 5.04 (ddt, *J* = 10.8, 7.9, 6.4 Hz, 1H), 3.88 (s, 3H), 3.36 (dd,

J = 17.6, 10.8 Hz, 1H), 3.16 – 3.07 (m, 2H), 2.96 (dd, *J* = 14.0, 6.6 Hz, 1H); ^{13}C NMR (101 MHz, $CDCl_3$) δ 184.7, 164.2, 157.9, 136.3, 132.9(2C), 129.6(2C), 128.8(2C), 127.1, 113.8(2C), 83.3, 55.7, 41.1, 38.8; IR νmax 3022, 2801, 1618, 1531, 802, 719, 676 cm^{-1}; HRMS (EI) *m/z* calcd for $C_{18}H_{18}NO_3$ [M + H]$^+$ 296.1281, found 296.1285.

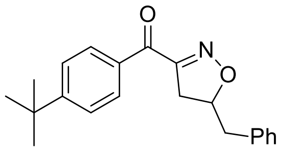

(5-Benzyl-4,5-dihydroisoxazol-3-yl)(4-tert-butylphenyl)methanone (3g)

The compound was prepared following the general procedure. Yield 63%. ^{1}H NMR (400 MHz, $CDCl_3$) δ 8.10 – 8.05 (m, 2H), 7.49 – 7.43 (m, 2H), 7.34 – 7.23 (m, 5H), 5.02 (ddt, *J* = 10.8, 7.9, 6.3 Hz, 1H), 3.32 (dd, *J* = 17.6, 10.8 Hz, 1H), 3.14 – 3.04 (m, 2H), 2.93 (dd, *J* = 14.0, 6.5 Hz, 1H), 1.34 (d, *J* = 4.4 Hz, 9H); ^{13}C NMR (101 MHz, $CDCl_3$) δ 186.0, 157.8, 157.4, 136.2, 133.3, 130.3(2C), 129.5(2C), 128.7(2C), 126.9, 125.4(2C), 83.4, 40.9, 38.4, 35.2, 31.1(3C); IR νmax 3030, 1660, 1601, 1403, 1375, 860, 750, 700 cm^{-1}; HRMS (EI) *m/z* calcd for $C_{21}H_{24}NO_2$ [M + H]$^+$ 322.1802, found 322.1808.

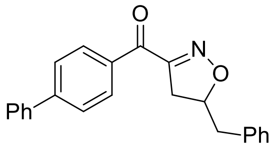

[1,1'-Biphenyl]-4-yl(5-benzyl-4,5-dihydroisoxazol-3-yl)methanone (3h).

The compound was prepared following the general procedure. Yield 57%. ^{1}H NMR (400 MHz, $CDCl_3$) δ 8.25 – 8.19 (m, 2H), 7.69 – 7.68 (m, 1H), 7.67 (m, 1H), 7.65 (m, 1H), 7.63 (m, 1H), 7.49 (m, 1H), 7.47 (m, 1H), 7.46 (m, 1H), 7.43 (m, 1H), 7.35 – 7.33 (m, 1H), 7.32 (m, 1H), 7.30 – 7.28 (m, 2H), 5.08 (ddt, *J* = 10.8, 7.9, 6.3 Hz, 1H), 3.38 (dd, *J* = 17.6, 10.8 Hz, 1H), 3.17 – 3.09 (m, 2H), 2.98 (dd, *J* = 14.0, 6.5 Hz, 1H); ^{13}C NMR (101 MHz, $CDCl_3$) δ 186.0, 157.9, 146.4, 139.9, 136.2, 134.6, 131.0(2C), 129.6(2C), 129.1(2C), 128.8(2C), 128.5(2C), 127.5(2C), 127.1(2C), 83.7, 41.1, 38.5; IR νmax 3029, 1655, 1648, 1401, 1362, 842, 746, 693 cm^{-1}; HRMS (EI) *m/z* calcd for $C_{23}H_{20}NO_2$ [M + H]$^+$ 342.1489, found 342.1483. These data are consistent with reported literature values[2].

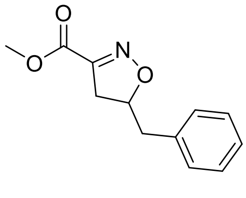

Methyl 5-benzyl-4,5-dihydroisoxazole-3-carboxylate（3i）

The compound was prepared following the general procedure. Yield 30%. ^{1}H NMR (400 MHz, $CDCl_3$) δ 7.32 (m, 2H), 7.26 (m, 1H), 7.22 (m, 2H), 5.06 (ddd, *J* = 14.7, 10.9, 6.7 Hz, 1H), 3.86 (s, 3H), 3.18 (dd, *J* = 17.7, 10.9 Hz, 1H), 3.11 (dd, *J* = 14.0, 6.1 Hz, 1H), 2.94 (dd, *J* = 14.8, 5.3 Hz, 1H), 2.92 – 2.85 (m, 1H); ^{13}C NMR (101 MHz, $CDCl_3$) δ 161.2, 151.3, 136.0, 129.5(2C), 128.8(2C), 127.1, 84.5, 52.8, 40.8, 37.9; IR νmax 3418, 3032, 1717, 1584, 1449, 1366, 1265, 1126, 949, 746, 702, 582 cm^{-1}; HRMS (EI) *m/z* calcd for $C_{12}H_{14}NO_3$ $[M + H]^+$ 220.0974, found 220.0980.

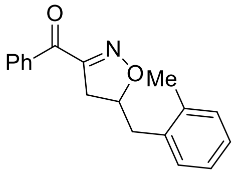

(5-(2-Methylbenzyl)-4,5-dihydroisoxazol-3-yl)(phenyl)methanone (5a)

The compounds was prepared following the general procedure as isomers. Yield 71%. ^{1}H NMR (400 MHz, $CDCl_3$) δ 8.30 – 8.23 (m, 2H), 7.66 (m, 1H), 7.57 – 7.49 (m, 2H), 7.30 – 7.23 (m, 4H), 5.19 – 5.08 (m, 1H), 3.42 (ddd, *J* = 17.5, 10.7, 0.9 Hz, 1H), 3.22 (dt, *J* = 12.6, 7.5 Hz, 2H), 2.99 (dd, *J* = 14.3, 6.7 Hz, 1H), 2.44 (s, 3H); ^{13}C NMR (101 MHz, $CDCl_3$) δ 186.5, 157.9, 136.6, 135.9, 134.6, 133.6, 130.6, 130.4(2C), 130.0, 128.4(2C), 127.1, 126.3, 82.9, 38.6, 38.1, 19.8; IR νmax 3028, 2940, 1660, 1570, 750, 690 cm^{-1}; HRMS (EI) *m/z* calcd for $C_{18}H_{18}NO_2$ $[M + H]^+$ 280.1332, found 280.1335.

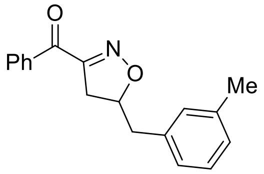

(5-(3-Methylbenzyl)-4,5-dihydroisoxazol-3-yl)(phenyl)methanone (5b)

The compound was prepared following the general procedure. Yield 66%. ^{1}H NMR (400 MHz, $CDCl_3$) δ 8.15 (d, *J* = 7.2 Hz, 2H), 7.59 (m, 1H), 7.47 (m, 2H), 7.22

(m, 1H), 7.09 (m, 3H), 5.12 – 5.00 (m, 1H), 3.35 (dd, *J* = 17.5, 10.8, 1H), 3.17 – 3.04 (m, 2H), 2.97 – 2.87 (m, 1H), 2.36 (s, 3H); ^{13}C NMR (101 MHz, $CDCl_3$) δ 186.6, 157.8, 138.4, 136.1, 135.9, 133.7, 130.4(2C), 130.4, 128.7, 128.5(2C), 127.9, 126.6, 83.8, 40.9, 38.5, 21.5; IR νmax 3048, 2908, 1661, 1581, 862, 750, 691 cm^{-1}; HRMS (EI) *m/z* calcd for $C_{18}H_{18}NO_2$ $[M + H]^+$ 280.1332, found 280.1340.

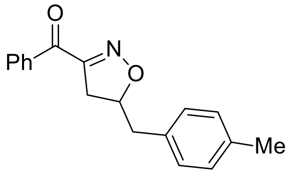

(5-(4-Methylbenzyl)-4,5-dihydroisoxazol-3-yl)(phenyl)methanone (5c)

The compound was prepared following the general procedure. Yield 64%. ^{1}H NMR (400 MHz, $CDCl_3$) δ 8.16 – 8.10 (m, 2H), 7.59 (m, 1H), 7.46 (m, 2H), 7.16 (m, 4H), 5.05 (dd, *J* = 11.0, 7.8 Hz, 1H), 3.35 (dd, *J* = 17.6, 10.8 Hz, 1H), 3.15 – 3.04 (m, 2H), 2.93 (dd, *J* = 14.0, 6.6 Hz, 1H), 2.34 (s, 3H); ^{13}C NMR (101 MHz, $CDCl_3$) δ 186.7, 157.9, 136.8, 136.1, 133.8, 133.2, 130.5(2C), 129.6(2C), 129.6(2C), 128.6(2C), 83.9, 40.7, 38.5, 21.3; IR νmax 3039, 2909, 1710, 1609, 822, 741, 680 cm^{-1}; HRMS (EI) *m/z* calcd for $C_{18}H_{18}NO_2$ $[M + H]^+$ 280.1332, found 280.1339.

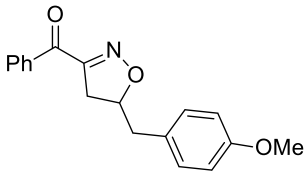

(5-(4-Methoxybenzyl)-4,5-dihydroisoxazol-3-yl)(phenyl)methanone (5d)

The compound was prepared following the general procedure. Yield 72%. ^{1}H NMR (400 MHz, $CDCl_3$) δ 8.12 (m, 2H), 7.58 (m, 1H), 7.45 (m, 2H), 7.18 (m, 2H), 6.86 (m, 2H), 5.08 – 4.96 (m, 1H), 3.78 (s, 3H), 3.34 (dd, *J* = 17.6, 10.8 Hz, 1H), 3.14 – 2.99 (m, 2H), 2.91 (dd, *J* = 14.1, 6.4 Hz, 1H); ^{13}C NMR (101 MHz, $CDCl_3$) δ 186.6, 158.7, 157.8, 135.9, 133.6, 130.6(2C), 130.4(2C), 128.4(2C), 128.1, 114.2(2C), 83.8, 55.3, 40.0, 38.3; IR νmax 3050, 2850, 1670, 1580, 820, 750, 691 cm^{-1}; HRMS (EI) *m/z* calcd for $C_{18}H_{18}NO_3$ $[M + H]^+$ 296.1281, found 296.1285.

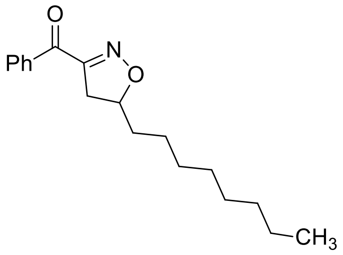

(5-Octyl-4,5-dihydroisoxazol-3-yl)(phenyl)methanone (5e)

The compound was prepared following the general procedure. Yield 89%. ^{1}H NMR (400 MHz, $CDCl_3$) δ 8.25 – 8.12 (m, 2H), 7.58 (m, 1H), 7.46 (m, 2H), 4.79 (ddt, *J* = 10.9, 8.4, 6.6 Hz, 1H), 3.39 (dd, *J* = 17.4, 10.9 Hz, 1H), 3.00 (dd, *J* = 17.4, 8.5 Hz, 1H), 1.79 (m, 1H), 1.69 – 1.57 (m, 1H), 1.45 – 1.19 (m, 12H), 0.88 (m, 3H); ^{13}C NMR (101 MHz, $CDCl_3$) δ 186.7, 157.9, 136.0, 133.6, 130.5(2C), 128.5(2C), 83.7, 38.9, 35.3, 31.9, 29.6, 29.5, 29.3, 25.4, 22.8, 14.2; IR νmax 3062, 2948, 1635, 1541, 760, 710 cm^{-1}; HRMS (EI) *m/z* calcd for $C_{18}H_{26}NO_2$ $[M + H]^+$ 288.1958, found 288.1961. These data are consistent with reported literature values[2].

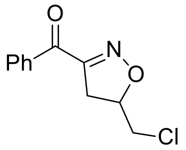

(5-(Chloromethyl)-4,5-dihydroisoxazol-3-yl)(phenyl)methanone (5f)

The compound was prepared following the general procedure. Yield 83%. ^{1}H NMR (400 MHz, $CDCl_3$) δ 8.24 – 8.14 (m, 2H), 7.66 – 7.55 (m, 1H), 7.53 – 7.44 (m, 2H), 5.05 (dddd, *J* = 11.1, 7.1, 5.7, 4.5 Hz, 1H), 3.76 – 3.62 (m, 2H), 3.50 (dd, *J* = 17.9, 11.1 Hz, 1H), 3.36 (dd, *J* = 17.9, 7.1 Hz, 1H); ^{13}C NMR (101 MHz, $CDCl_3$) δ 186.0, 157.5, 135.7, 133.9, 130.5(2C), 128.6(2C), 81.2, 45.1, 37.7; IR νmax 3025, 1660, 1580, 700 cm^{-1}; HRMS (EI) *m/z* calcd for $C_{11}H_{11}NO_2Cl$ $[M + H]^+$ 224.0473, found 224.0466. These data are consistent with reported literature values[2].

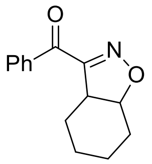

(3a,4,5,6,7,7a-Hexahydrobenzo[d]isoxazol-3-yl)(phenyl)methanone (5g)

The compound was prepared following the general procedure. Yield 54%. ^{1}H NMR (400 MHz, $CDCl_3$) δ 8.18 (m, 2H), 7.61 – 7.56 (m, 1H), 7.47 (m, 2H), 4.59 (dt,

J = 7.9, 3.9 Hz, 1H), 3.46 – 3.38 (m, 1H), 2.20 (dd, J = 15.2, 3.5 Hz, 1H), 2.11 – 2.01 (m, 1H), 1.82 (tt, J = 15.3, 4.7 Hz, 1H), 1.65 – 1.52 (m, 3H), 1.35 – 1.24 (m, 2H); ^{13}C NMR (101 MHz, $CDCl_3$) δ 187.0, 163.8, 136.4, 133.6, 130.4(2C), 128.5(2C), 82.3, 44.3, 25.6, 25.0, 21.7, 19.9; IR νmax 3060, 2940, 1660, 1550, 747, 704 cm^{-1}; HRMS (EI) *m/z* calcd for $C_{14}H_{16}NO_2$ [M + H]$^+$ 230.1176, found 230.1182. These data are consistent with reported literature values[2].

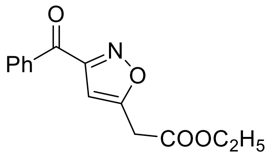

Ethyl 3-benzoyl-4,5-dihydroisoxazole-5-carboxylate (7a)

The compound was prepared following the general procedure. Yield 68%. ^{1}H NMR (400 MHz, $CDCl_3$) δ 8.33 – 8.28 (m, 2H), 7.71 – 7.65 (m, 1H), 7.59 – 7.51 (m, 2H), 7.43 (s, 1H), 4.48 (q, J = 7.1 Hz, 2H), 1.44 (t, J = 7.1 Hz, 3H); ^{13}C NMR (101 MHz, $CDCl_3$) δ 184.7, 162.3, 161.3, 156.4, 135.3, 134.6, 130.9(2C), 128.9(2C), 110.2, 62.8, 14.3; IR νmax 3058, 2945, 1655, 1545, 740, 702 cm^{-1}; HRMS (EI) *m/z* calcd for $C_{13}H_{14}NO_2$ [M + H]$^+$ 248.0917, found 248.0912.

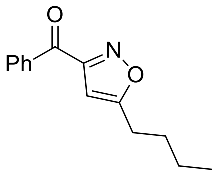

(5-Butylisoxazol-3-yl)(phenyl)methanone (7b).

The compound was prepared following the general procedure. Yield 64%. ^{1}H NMR (400 MHz, $CDCl_3$) δ 8.29 (m, 2H), 7.61 (m, 1H), 7.49 (m, 2H), 6.51 (ms, 1H), 2.82 (t, J = 7.6 Hz, 2H), 1.73 (dt, J = 15.2, 7.5 Hz, 2H), 1.42 (dq, J = 14.6, 7.4 Hz, 2H), 0.95 (t, J = 7.4 Hz, 3H); ^{13}C NMR (101 MHz, $CDCl_3$) δ 186.2, 174.8, 161.9, 135.9, 133.9, 130.7(2C), 128.6(2C), 101.7, 29.6, 26.4, 22.3, 13.8. IR νmax 3075, 2950, 2875, 1670, 1590, 740, 690 cm^{-1}; HRMS (EI) *m/z* calcd for $C_{14}H_{16}NO_2$ [M + H]$^+$ 230.1176, found 230.1171. These data are consistent with reported literature values[2].

References

1. Lee, J. J.; Kim, J.; Jun, Y. M.; Lee, B. M.; Kim, B. H., Indium-mediated one-pot synthesis of benzoxazoles or oxazoles from 2-nitrophenols or 1-aryl-2-nitroethanones. *Tetrahedron* **2009,** *65* (43), 8821-8831.

2. Itoh, K.-i.; Aoyama, T.; Satoh, H.; Fujii, Y.; Sakamaki, H.; Takido, T.; Kodomari, M., Application of silica gel-supported polyphosphoric acid (PPA/SiO2) as a reusable solid acid catalyst to the synthesis of 3-benzoylisoxazoles and isoxazolines. *Tetrahedron Letters* **2011,** *52* (51), 6892-6895.

^{1}H and ^{13}C NMR spectra

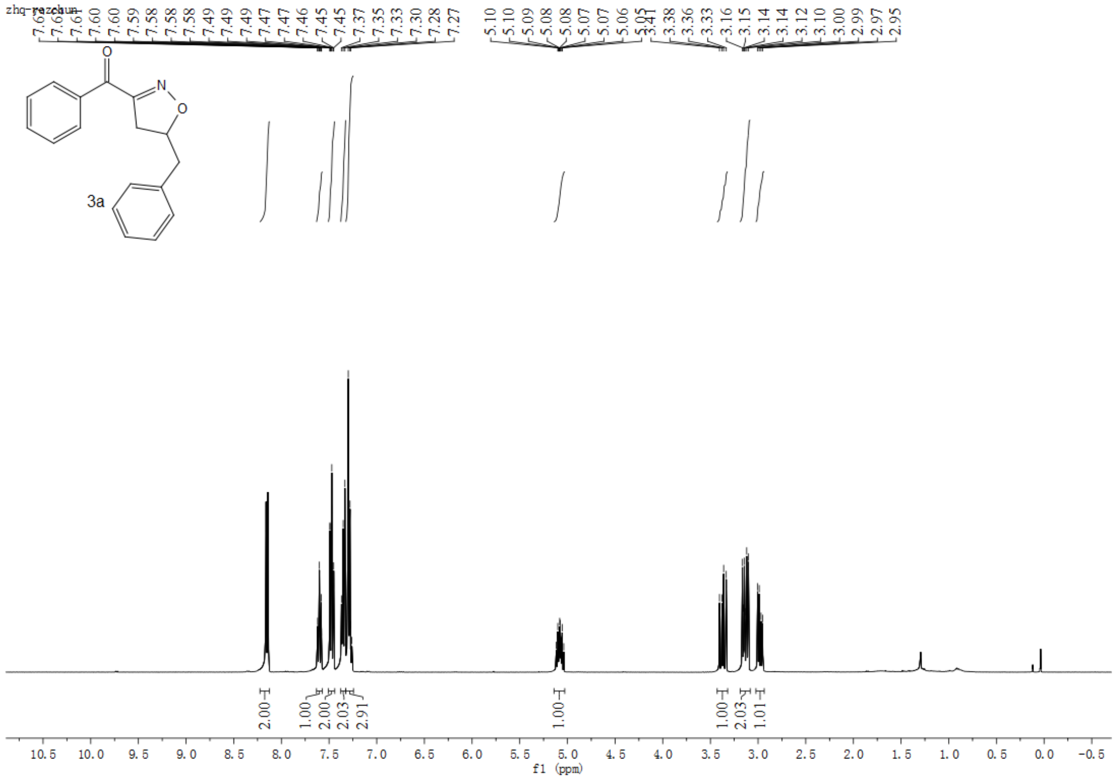

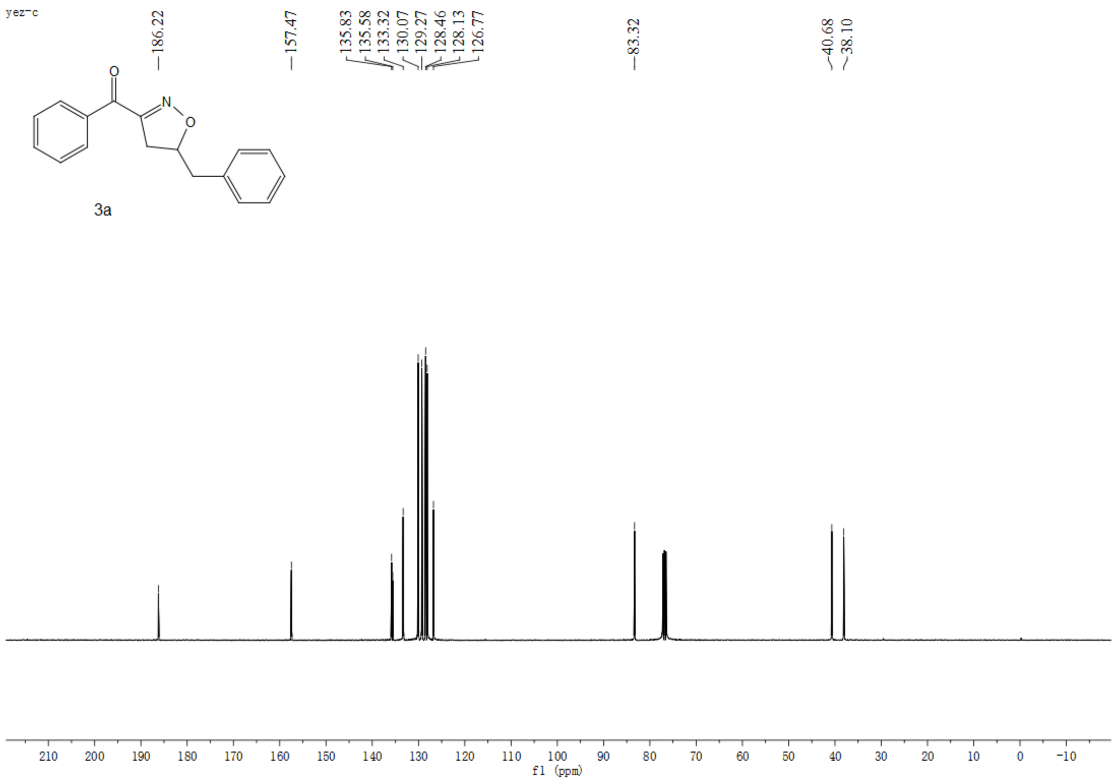
yez-c
186.22
157.47
135.83
135.58
133.32
130.07
129.27
128.46
128.13
126.77
83.32
40.68
38.10
3a
f1 (ppm)

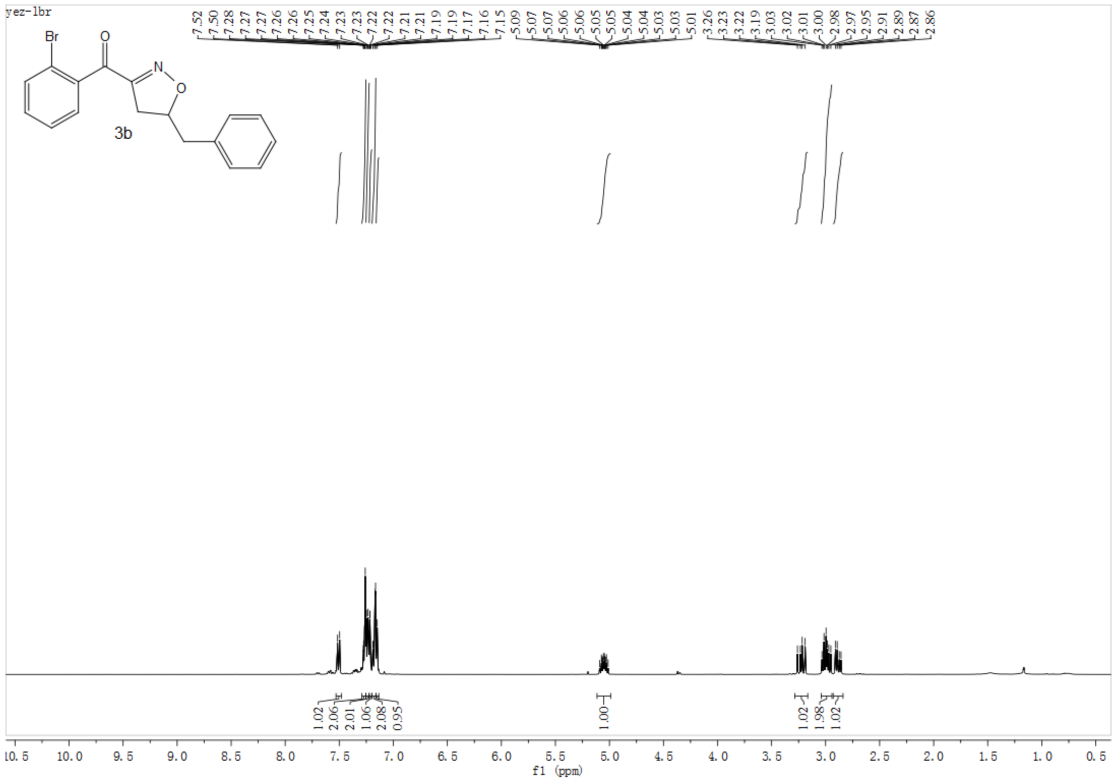
yez-1br
Br
3b
7.52
7.50
7.28
7.27
7.27
7.26
7.26
7.25
7.24
7.23
7.23
7.22
7.22
7.22
7.21
7.21
7.19
7.19
7.17
7.16
7.15
5.09
5.07
5.07
5.06
5.06
5.05
5.05
5.04
5.04
5.03
5.03
5.01
3.26
3.23
3.22
3.19
3.03
3.02
3.01
3.00
2.98
2.97
2.95
2.91
2.89
2.87
2.86
1.02
2.06
2.01
1.06
2.08
0.95
1.00
1.02
1.98
1.02
f1 (ppm)

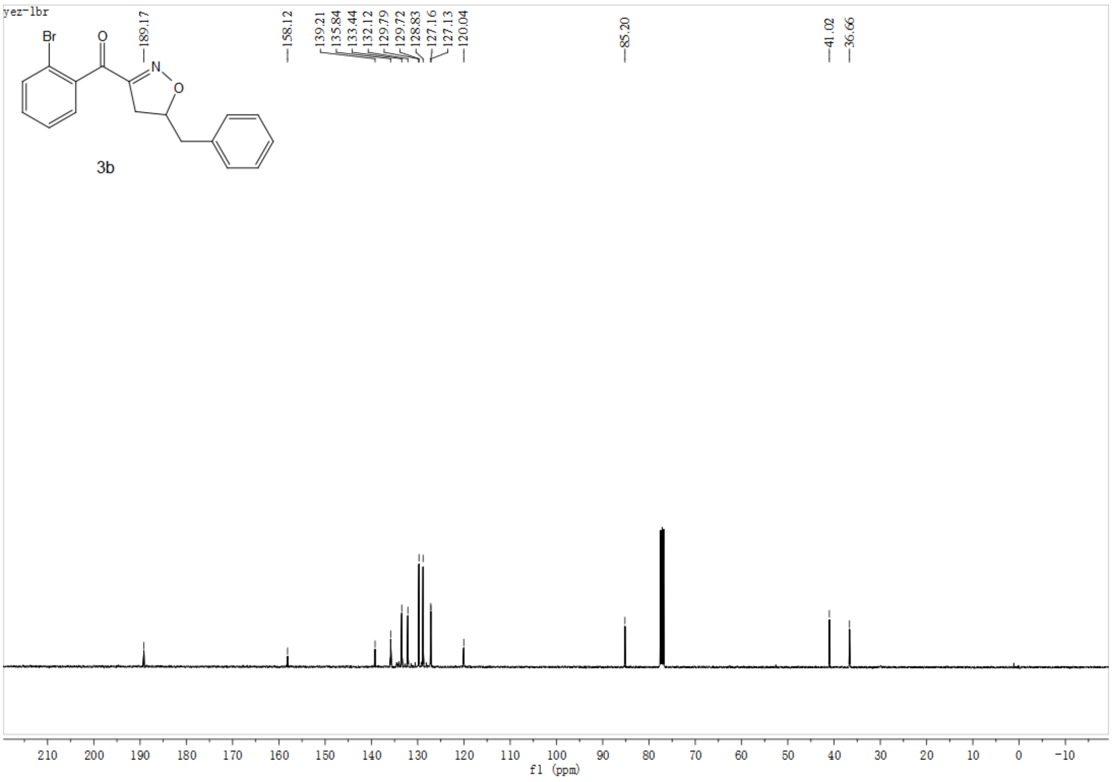
yez-1br
3b
f1 (ppm)

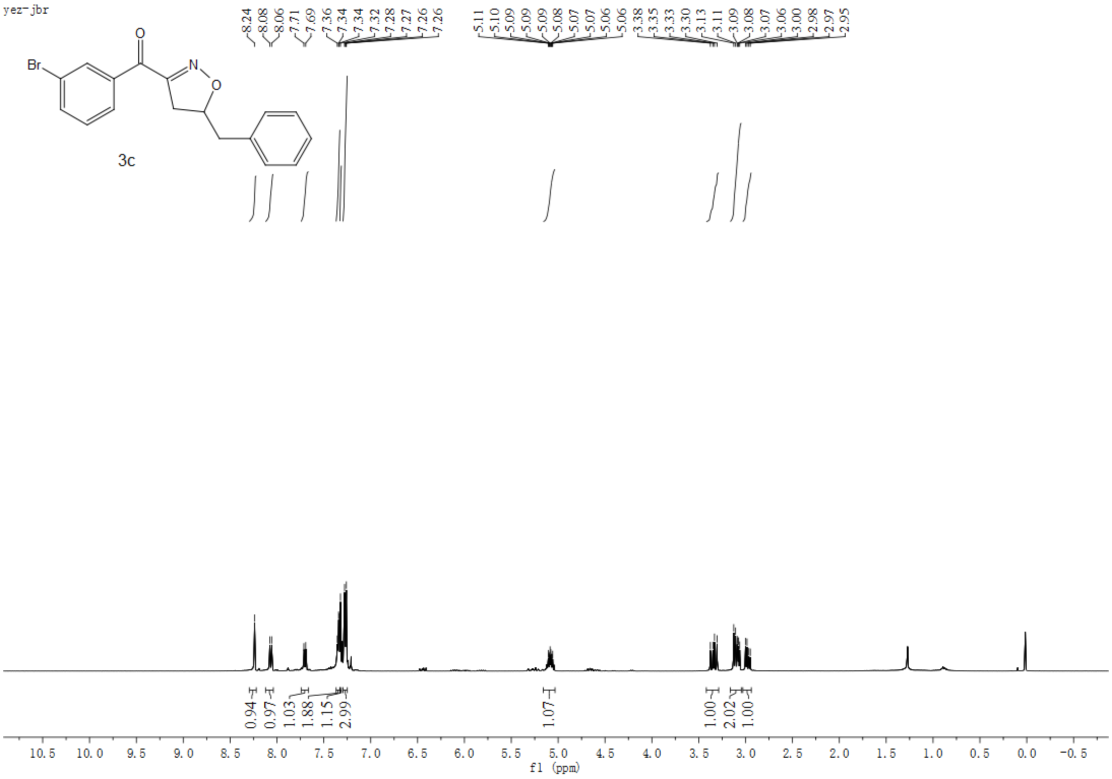
yez-jbr
3c
f1 (ppm)

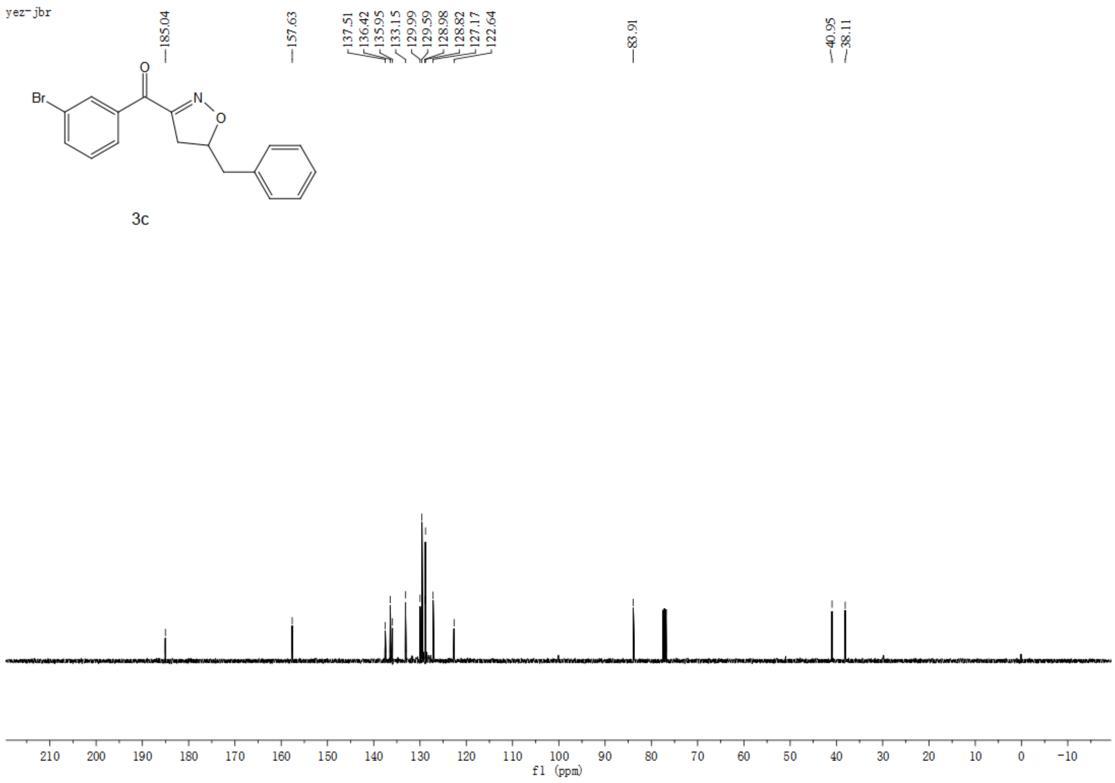

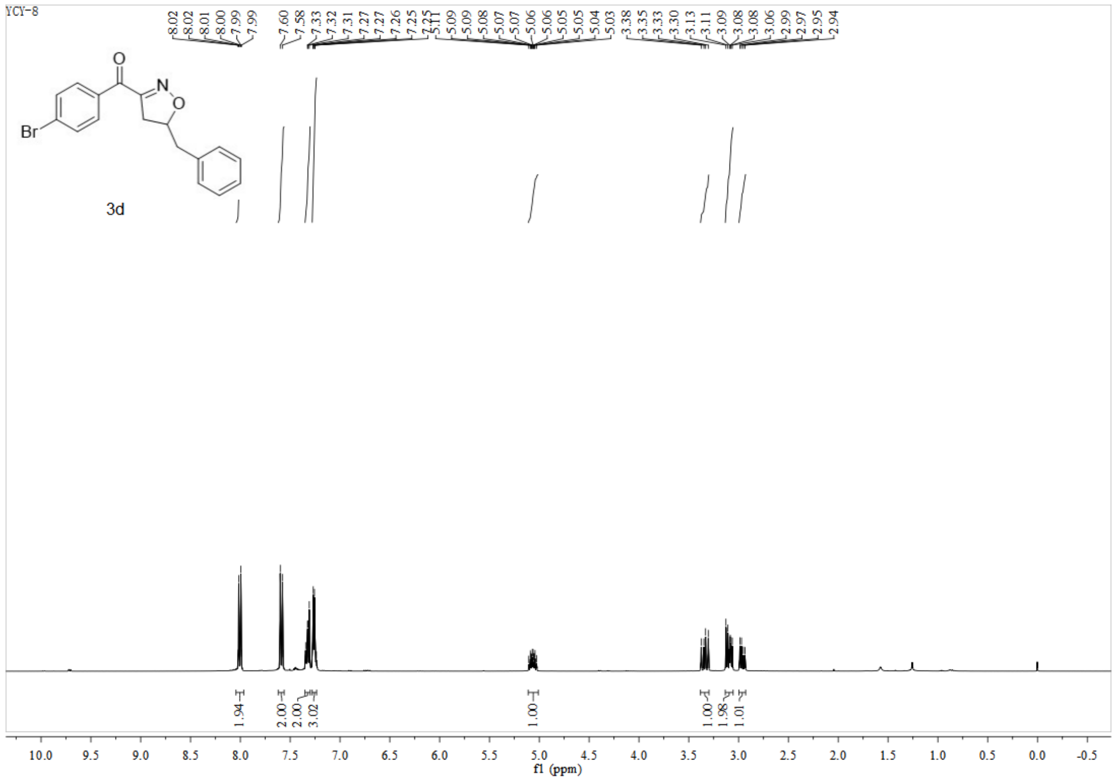

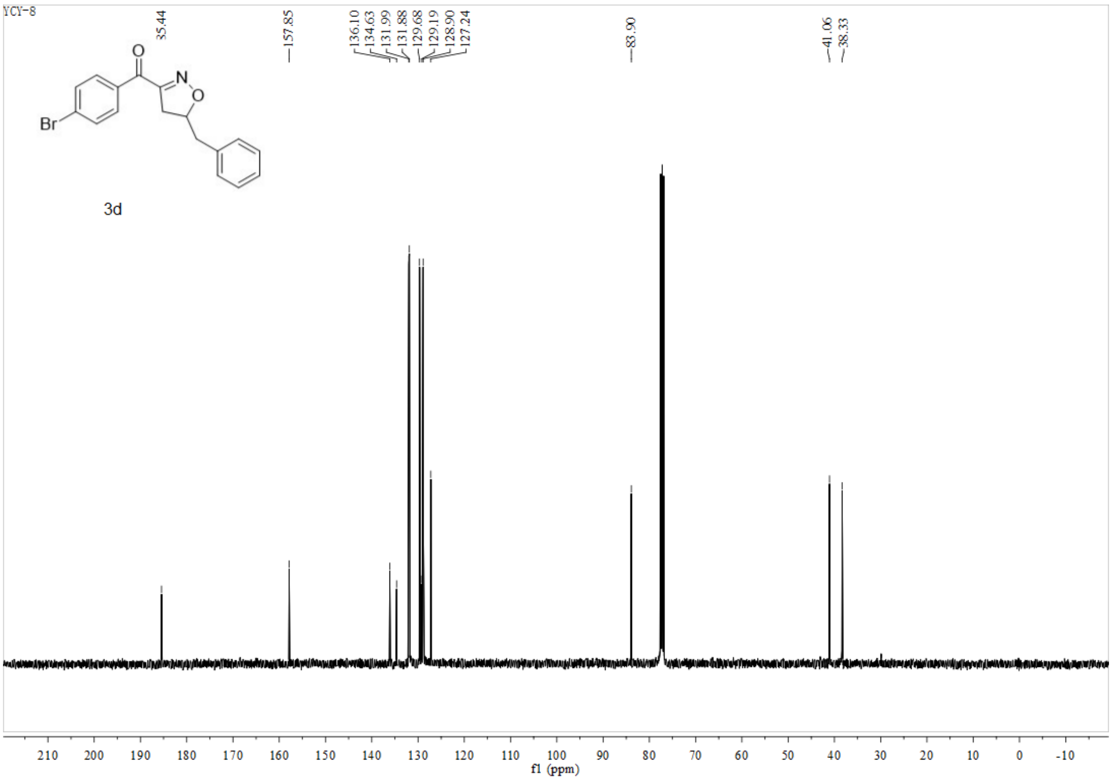
YCY-8
35.44
157.85
136.10
134.63
131.99
131.88
129.68
129.19
128.90
127.24
83.90
41.06
38.33
O
N
O
Br
3d
210 200 190 180 170 160 150 140 130 120 110 100 90 80 70 60 50 40 30 20 10 0 -10
f1 (ppm)

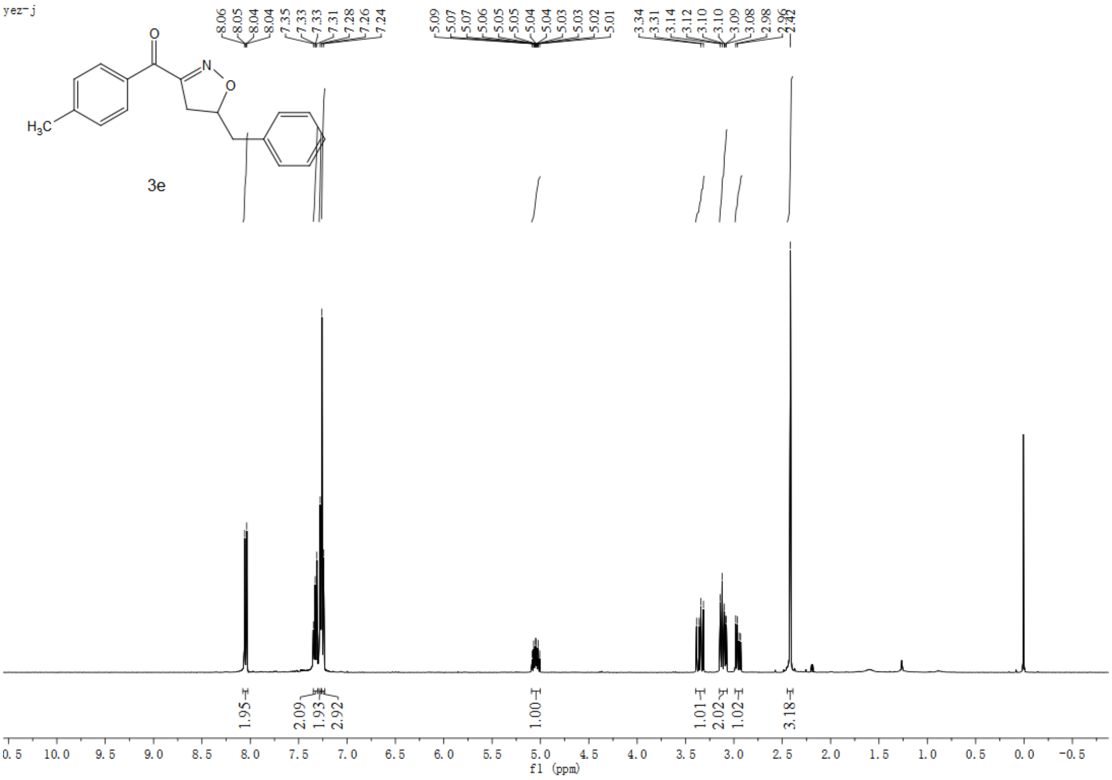
yez-j
8.06
8.05
8.04
8.04
7.35
7.33
7.33
7.31
7.28
7.26
7.24
5.09
5.07
5.07
5.06
5.05
5.05
5.04
5.04
5.03
5.03
5.02
5.01
3.34
3.31
3.14
3.12
3.10
3.10
3.09
3.08
2.98
2.96
2.42
O
N
O
H_3C
3e
1.95
2.09
1.93
2.92
1.00
1.01
2.02
1.02
3.18
0.5 10.0 9.5 9.0 8.5 8.0 7.5 7.0 6.5 6.0 5.5 5.0 4.5 4.0 3.5 3.0 2.5 2.0 1.5 1.0 0.5 0.0 -0.5
f1 (ppm)

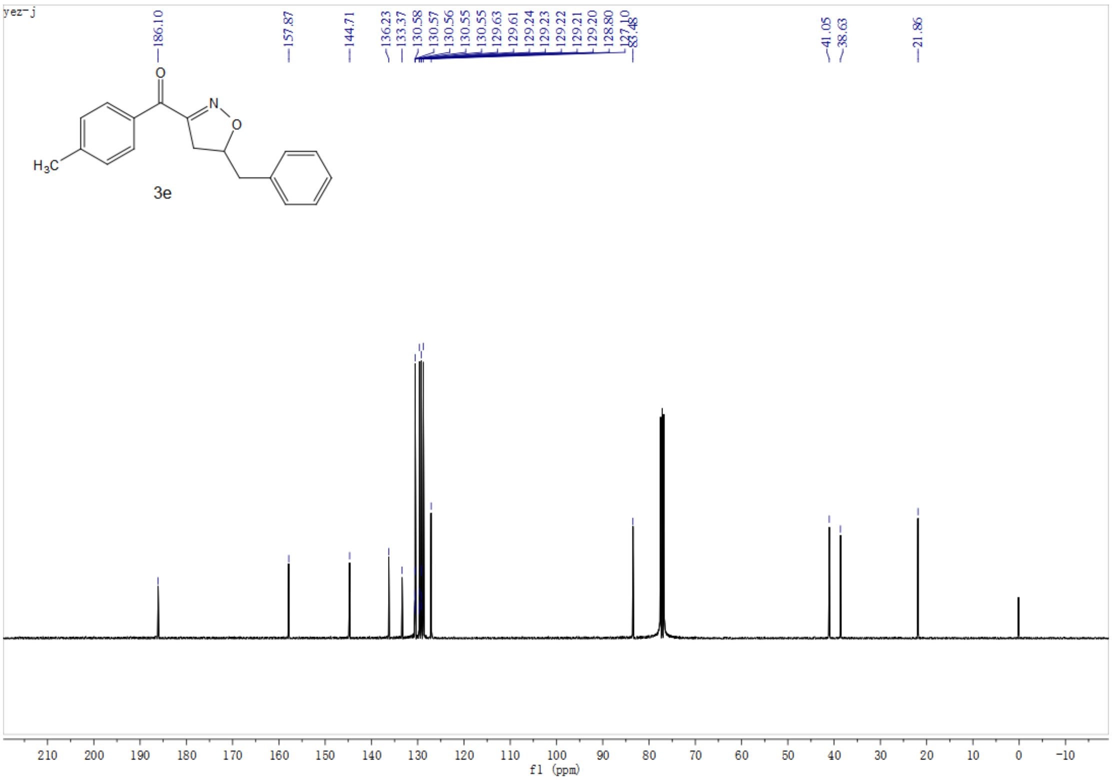
yez-j
186.10
157.87
144.71
136.23
133.37
130.58
130.57
130.56
130.55
130.55
129.63
129.61
129.24
129.23
129.22
129.21
129.20
128.80
127.10
83.48
41.05
38.63
21.86
O
N
O
H_3C
3e
210 200 190 180 170 160 150 140 130 120 110 100 90 80 70 60 50 40 30 20 10 0 -10
f1 (ppm)

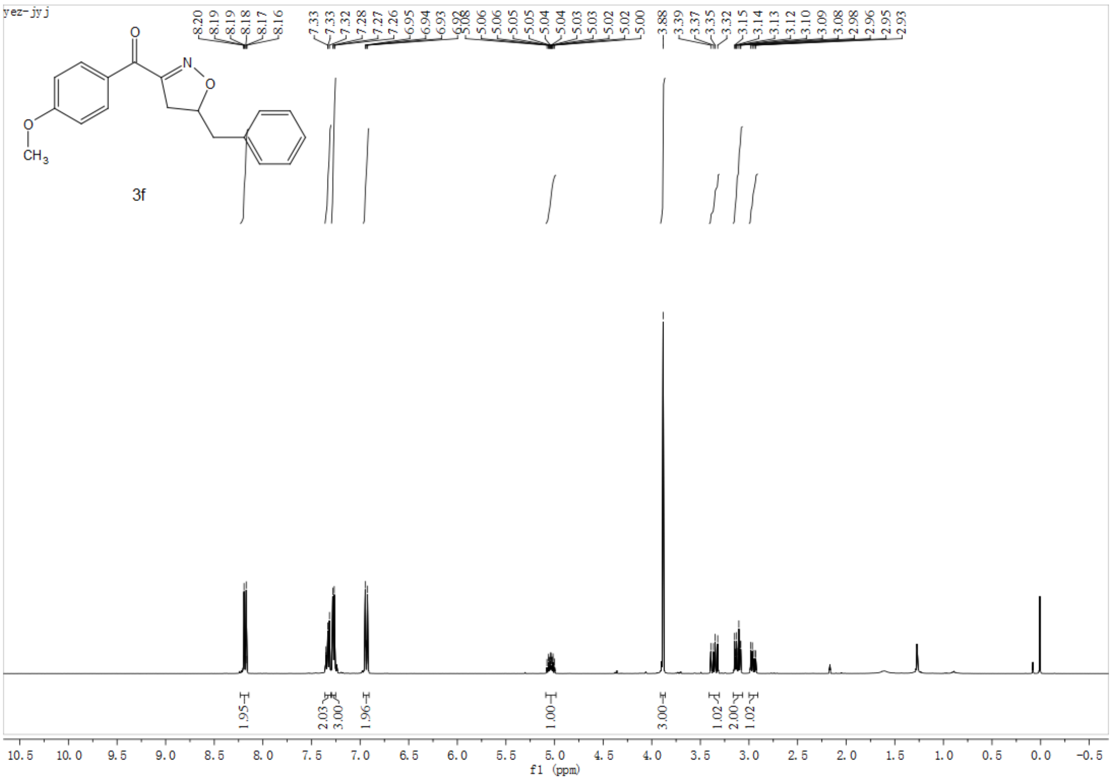
yez-jyj
8.20
8.19
8.19
8.18
8.17
8.16
7.33
7.33
7.32
7.28
7.27
7.26
6.95
6.94
6.93
6.92
5.08
5.06
5.06
5.05
5.05
5.04
5.04
5.03
5.03
5.02
5.02
5.00
3.88
3.39
3.37
3.35
3.32
3.15
3.14
3.13
3.12
3.10
3.09
3.08
2.98
2.96
2.95
2.93
O
N
O
O
CH_3
3f
1.95
2.03
3.00
1.96
1.00
3.00
1.02
2.00
1.02
10.5 10.0 9.5 9.0 8.5 8.0 7.5 7.0 6.5 6.0 5.5 5.0 4.5 4.0 3.5 3.0 2.5 2.0 1.5 1.0 0.5 0.0 -0.5
f1 (ppm)

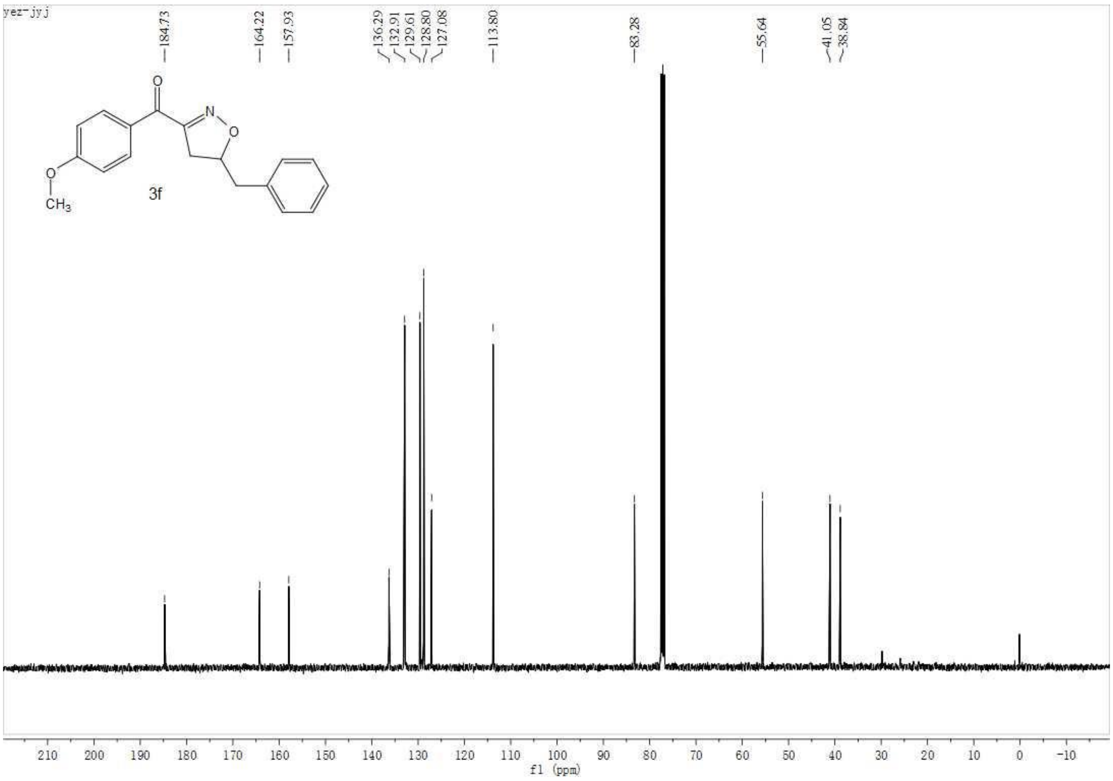
yez-jyj
184.73
164.22
157.93
136.29
132.91
129.61
128.80
127.08
113.80
83.28
55.64
41.05
38.84
O
N
O
O
CH3
3f
210 200 190 180 170 160 150 140 130 120 110 100 90 80 70 60 50 40 30 20 10 0 -10
f1 (ppm)

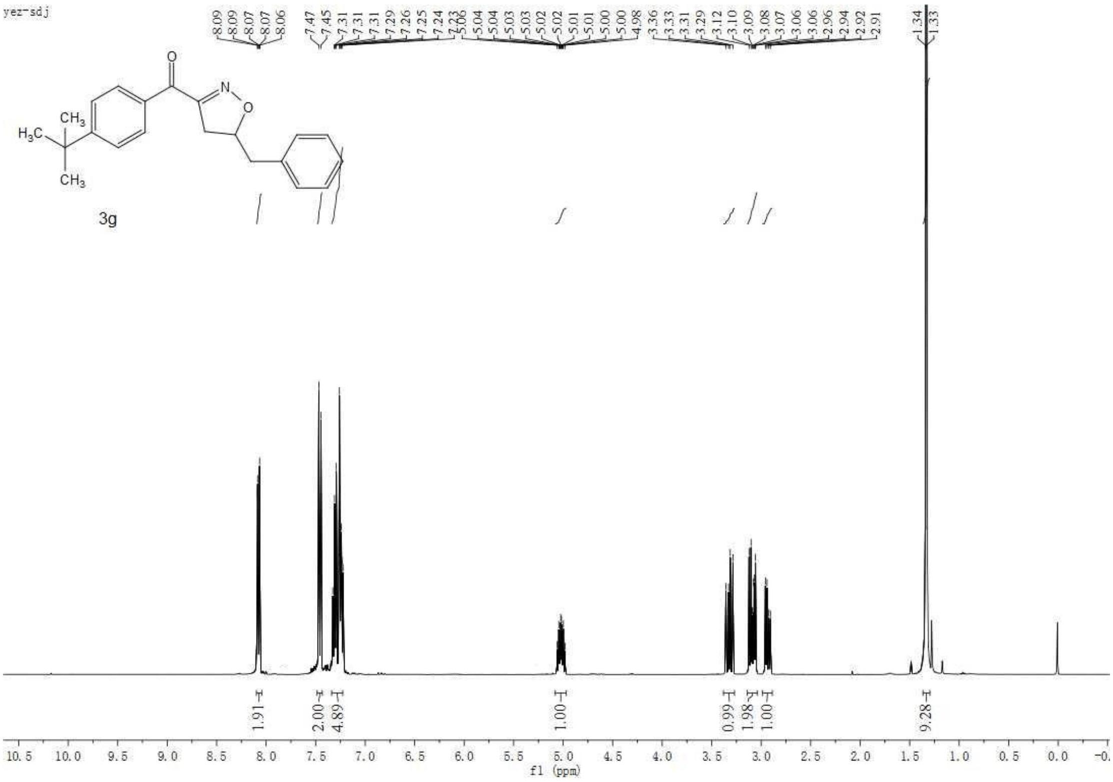
yez-sdj
8.09 8.09 8.07 8.07 8.06
7.47 7.45 7.31 7.31 7.31 7.29 7.26 7.25 7.24 7.23
5.06 5.04 5.04 5.03 5.03 5.02 5.02 5.01 5.01 5.00 5.00 4.98
3.36 3.33 3.31 3.29 3.12 3.10 3.09 3.08 3.07 3.06 3.06 2.96 2.94 2.92 2.91
1.34 1.33
O
N
O
CH3
H3C
CH3
3g
1.91
2.00
4.89
1.00
0.99
1.98
1.00
9.28
10.5 10.0 9.5 9.0 8.5 8.0 7.5 7.0 6.5 6.0 5.5 5.0 4.5 4.0 3.5 3.0 2.5 2.0 1.5 1.0 0.5 0.0
f1 (ppm)

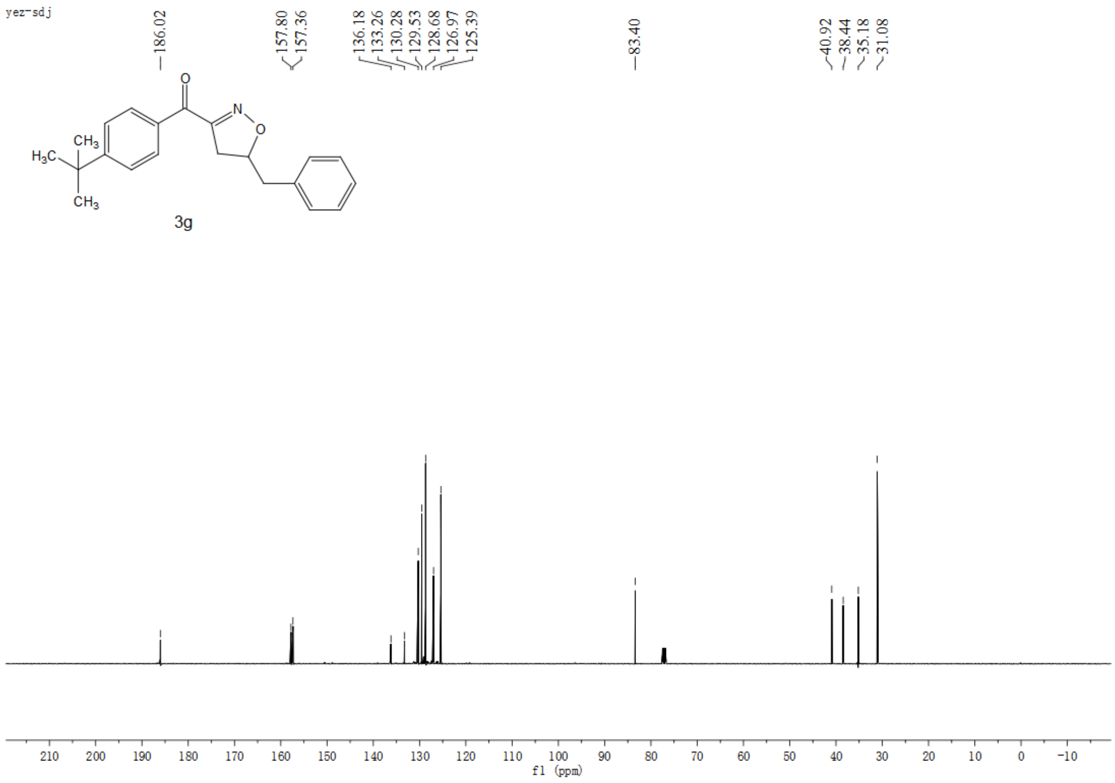
yez-sdj
186.02
157.80
157.36
136.18
133.26
130.28
129.53
128.68
126.97
125.39
83.40
40.92
38.44
35.18
31.08
3g
f1 (ppm)

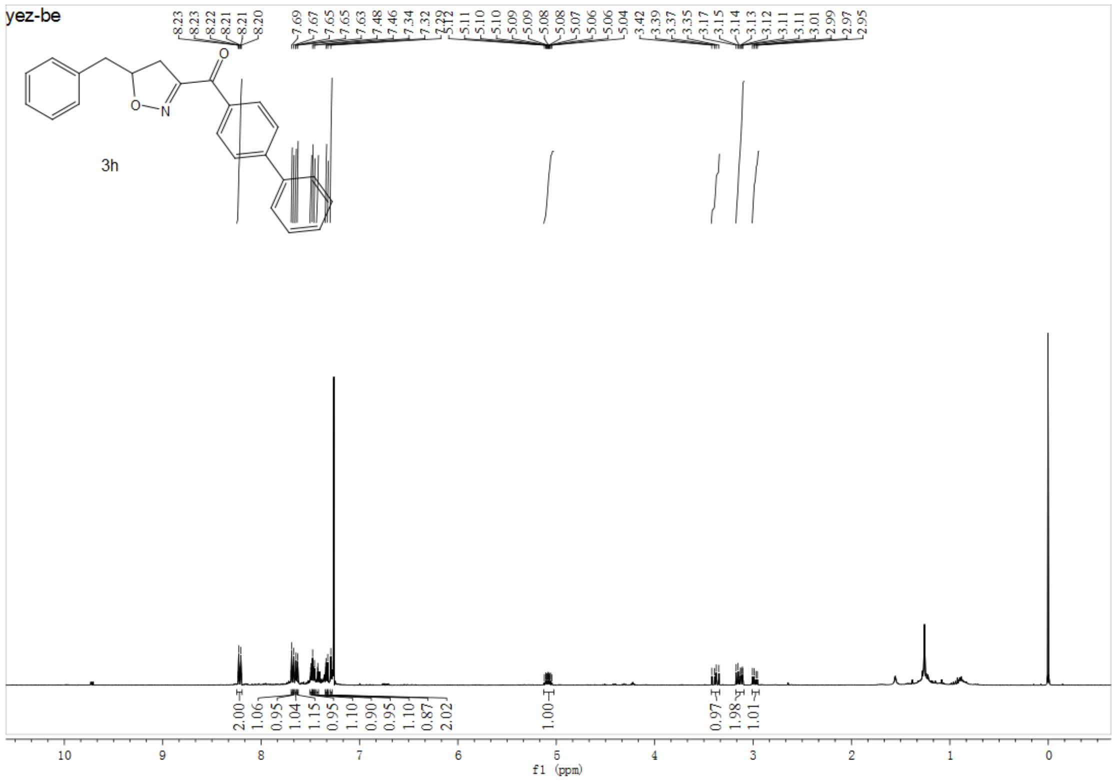
yez-be
8.23
8.23
8.22
8.21
8.21
8.20
7.69
7.67
7.65
7.65
7.63
7.48
7.46
7.34
7.32
7.29
7.12
5.11
5.10
5.10
5.09
5.09
5.08
5.08
5.07
5.06
5.06
5.04
3.42
3.39
3.37
3.35
3.17
3.15
3.14
3.13
3.12
3.11
3.11
3.01
2.99
2.97
2.95
3h
2.00
1.06
0.95
1.04
1.15
0.95
1.10
0.90
0.95
1.10
0.87
2.02
1.00
0.97
1.98
1.01
f1 (ppm)

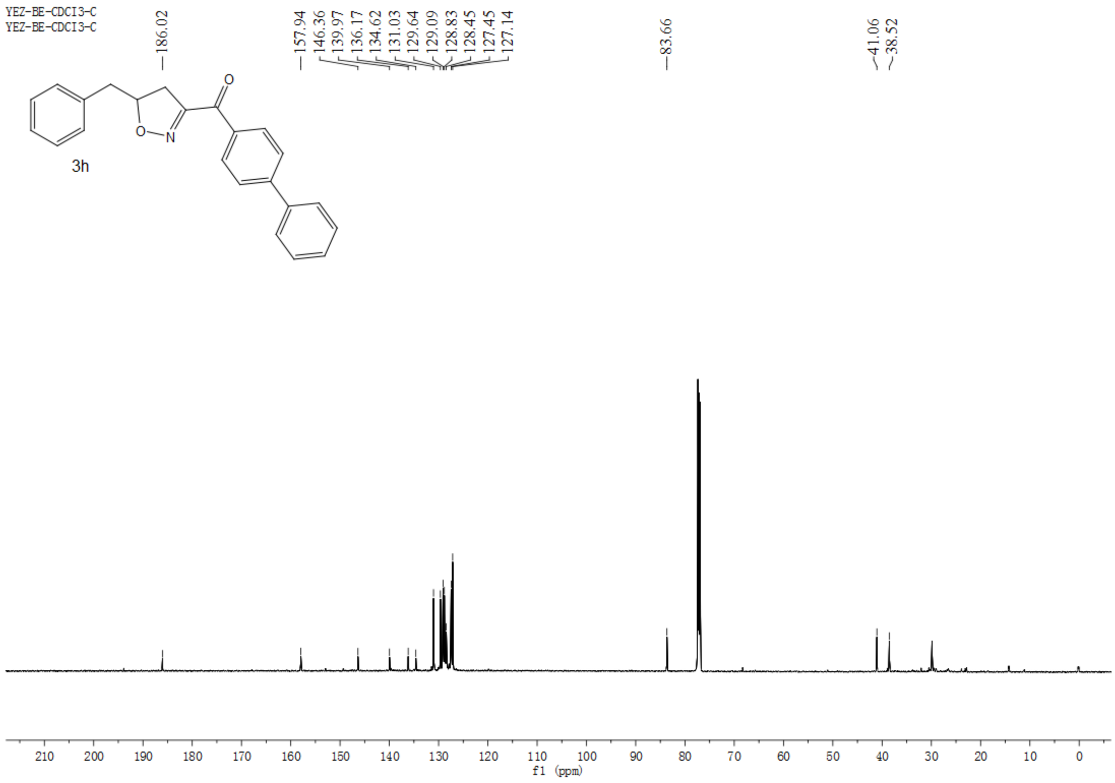
YEZ-BE-CDCl3-C
YEZ-BE-CDCl3-C
186.02
157.94
146.36
139.97
136.17
134.62
131.03
129.64
129.09
128.83
128.45
127.45
127.14
83.66
41.06
38.52
O
O–N
3h
210 200 190 180 170 160 150 140 130 120 110 100 90 80 70 60 50 40 30 20 10 0
f1 (ppm)

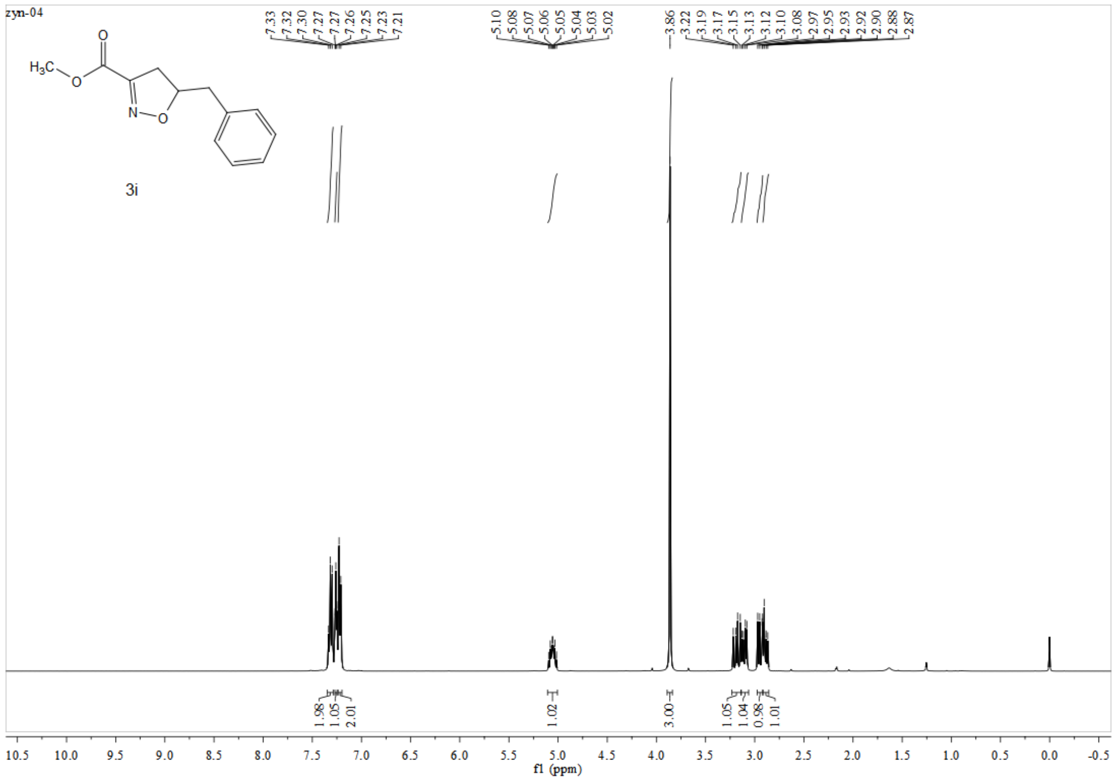
zyn-04
7.33
7.32
7.30
7.27
7.27
7.26
7.25
7.23
7.21
5.10
5.08
5.07
5.06
5.05
5.04
5.03
5.02
3.86
3.22
3.19
3.17
3.15
3.13
3.12
3.10
3.08
2.97
2.95
2.93
2.92
2.90
2.88
2.87
O
H_3C
O
N–O
3i
1.98
1.05
2.01
1.02
3.00
1.05
1.04
0.98
1.01
10.5 10.0 9.5 9.0 8.5 8.0 7.5 7.0 6.5 6.0 5.5 5.0 4.5 4.0 3.5 3.0 2.5 2.0 1.5 1.0 0.5 0.0 -0.5
f1 (ppm)

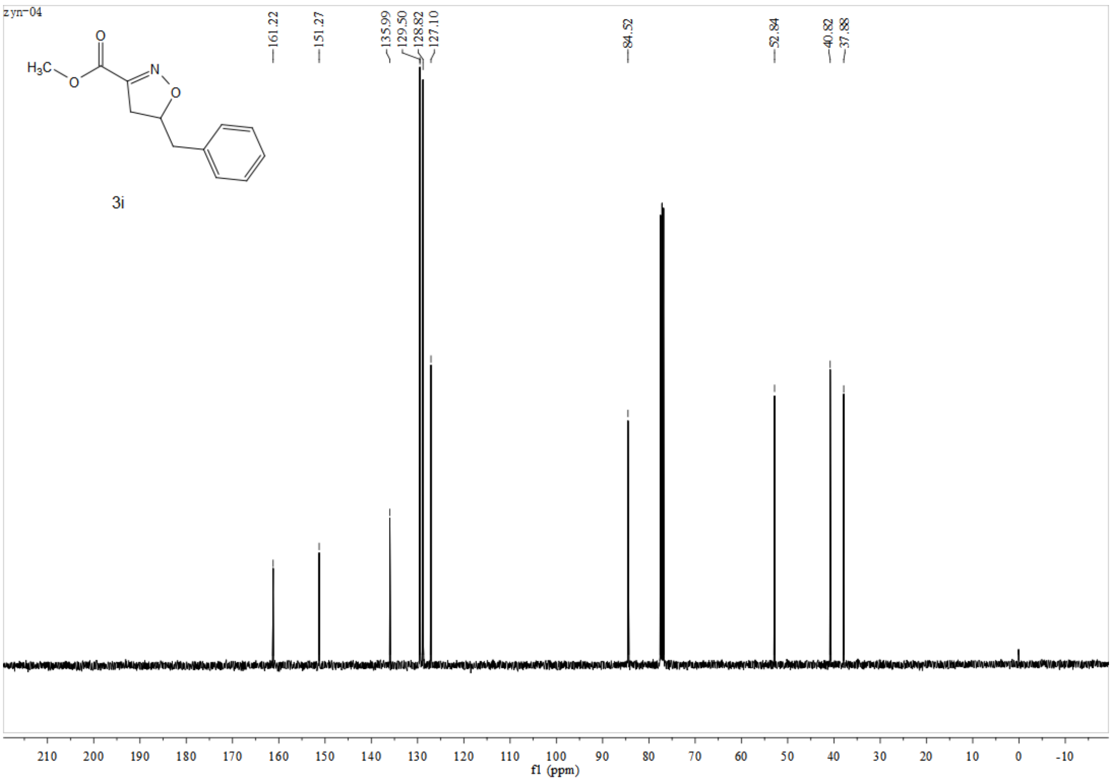
zyn-04
3i
161.22
151.27
135.99
129.50
128.82
127.10
84.52
52.84
40.82
37.88
f1 (ppm)

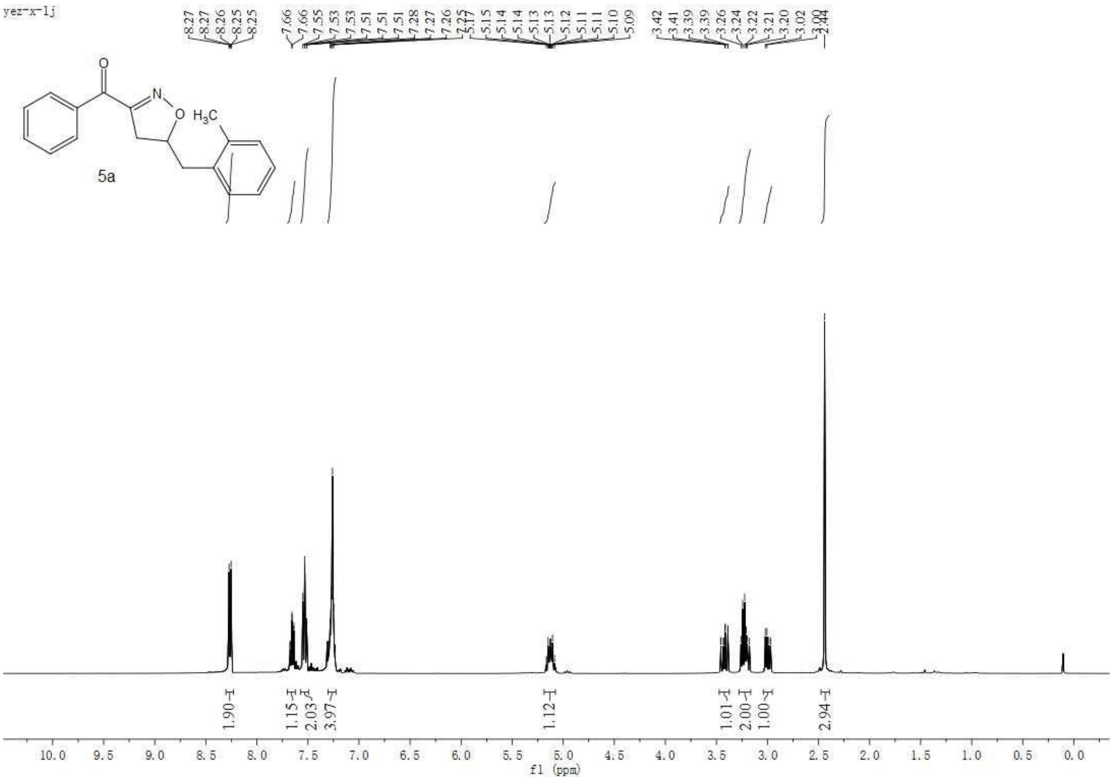
yez-x-1j
5a
1.90
1.15
2.03
3.97
1.12
1.01
2.00
1.00
2.94
f1 (ppm)

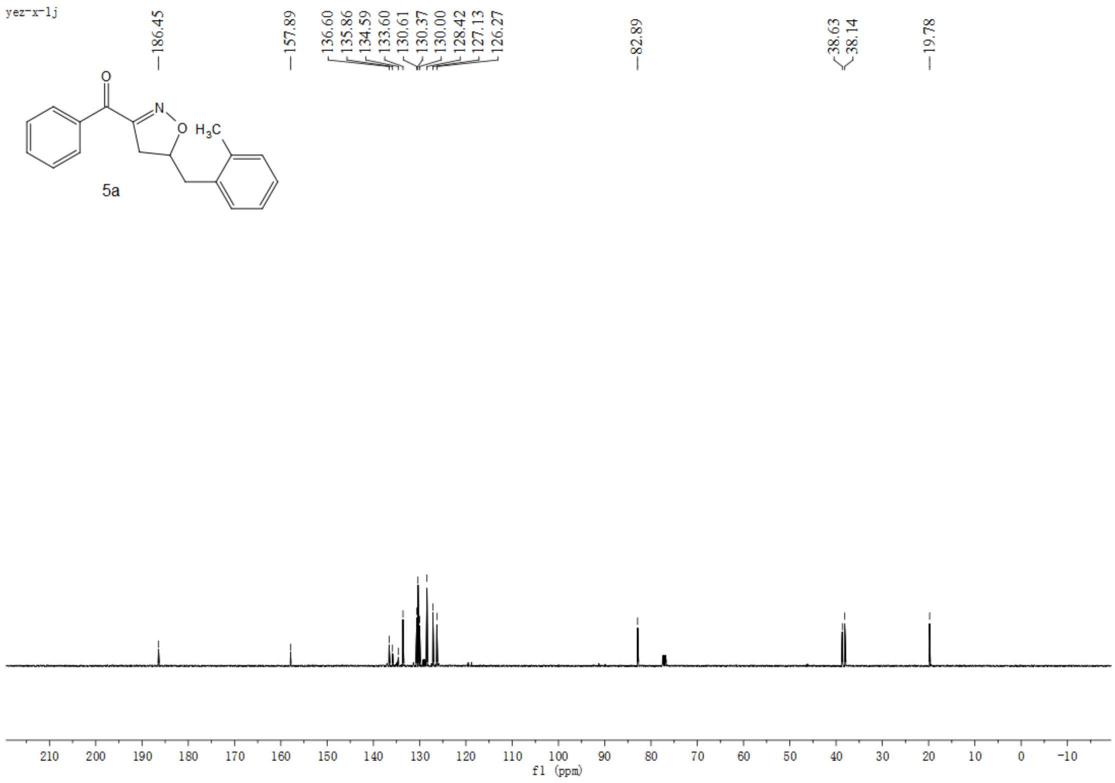
yez-x-1j
186.45
157.89
136.60
135.86
134.59
133.60
130.61
130.37
130.00
128.42
127.13
126.27
82.89
38.63
38.14
19.78
5a
f1 (ppm)

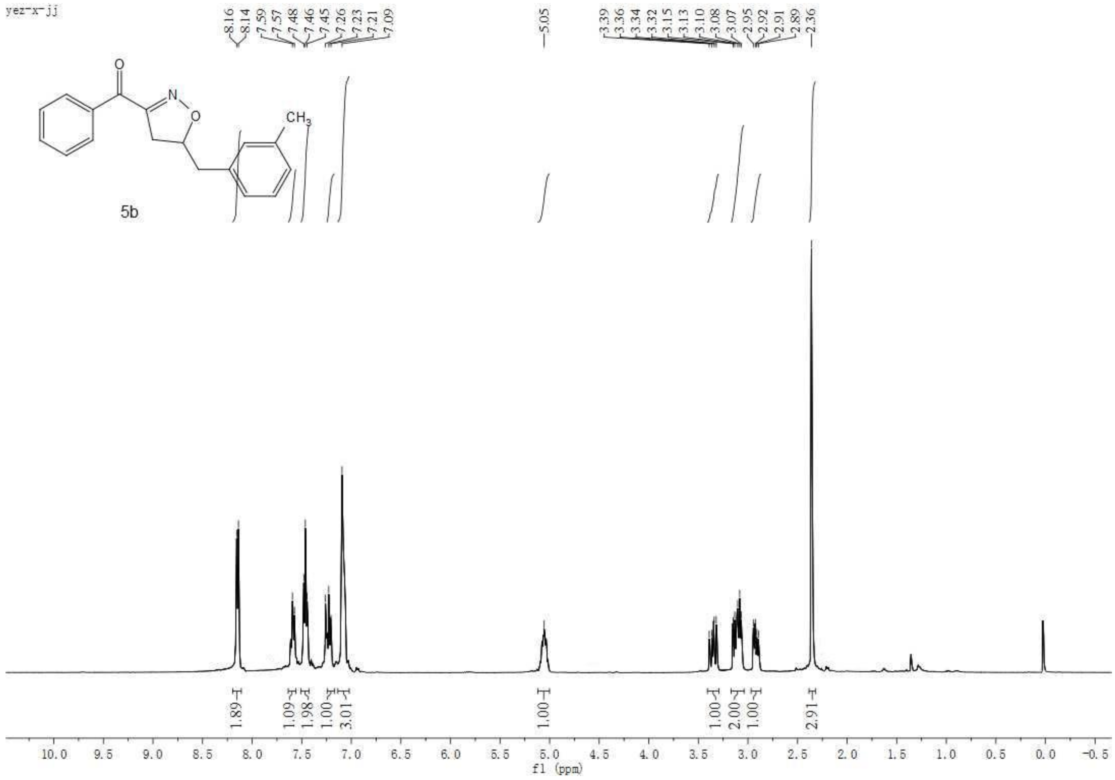
yez-x-jj
8.16
8.14
7.59
7.57
7.48
7.46
7.45
7.26
7.23
7.21
7.09
5.05
3.39
3.36
3.34
3.32
3.15
3.13
3.10
3.08
3.07
2.95
2.92
2.91
2.89
2.36
5b
1.89
1.09
1.98
1.00
3.01
1.00
1.00
2.00
1.00
2.91
f1 (ppm)

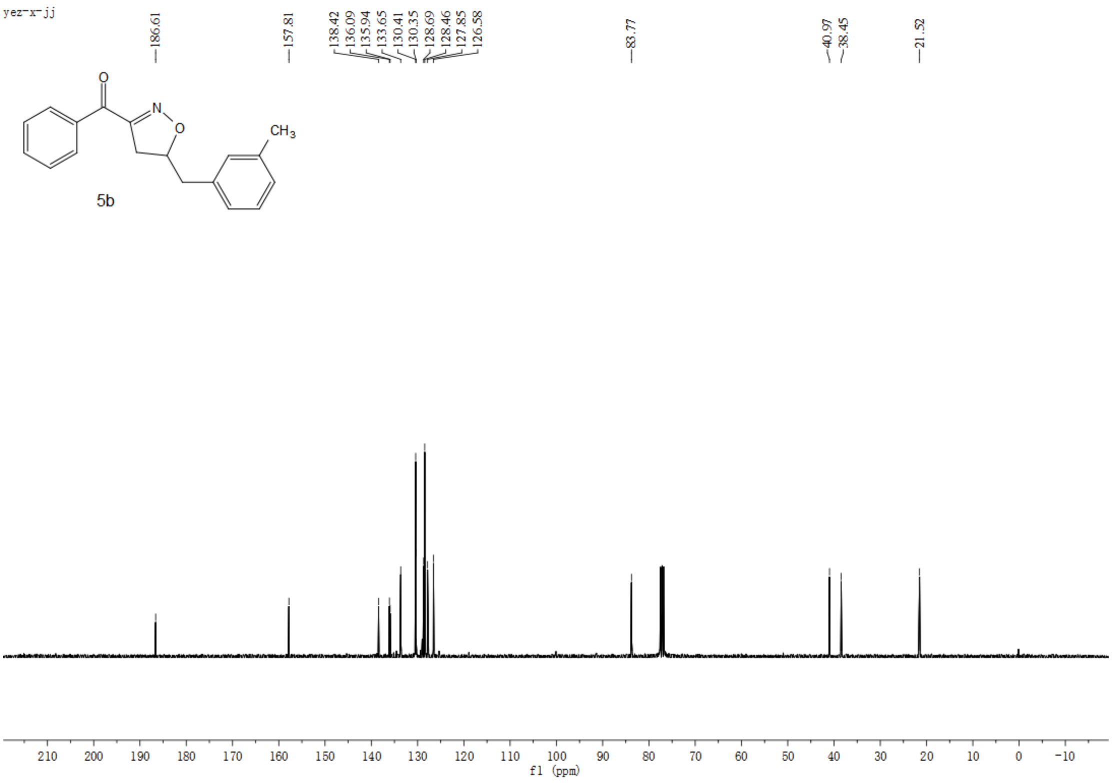
yez-x-jj
186.61
157.81
138.42
136.09
135.94
133.65
130.41
130.35
128.69
128.46
127.85
126.58
83.77
40.97
38.45
21.52
O
N
O
CH3
5b
210 200 190 180 170 160 150 140 130 120 110 100 90 80 70 60 50 40 30 20 10 0 -10
f1 (ppm)

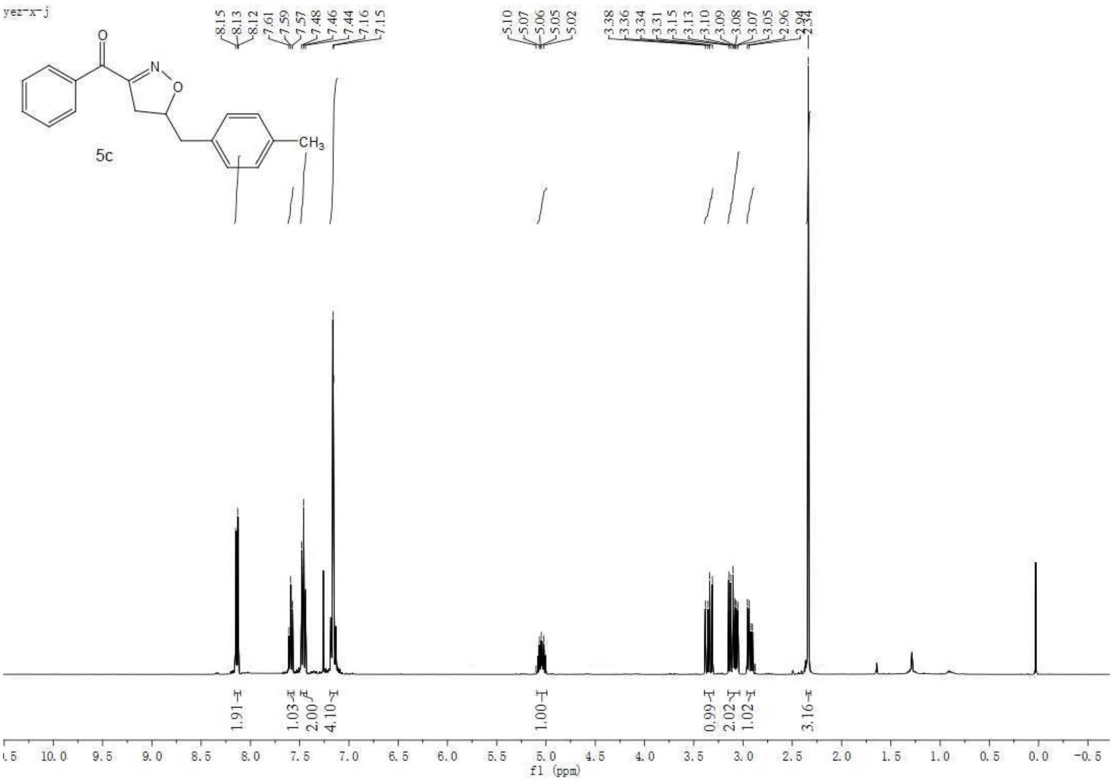
yez-x-j
8.15
8.13
8.12
7.61
7.59
7.57
7.48
7.46
7.44
7.16
7.15
5.10
5.07
5.06
5.05
5.02
3.38
3.36
3.34
3.31
3.15
3.13
3.10
3.09
3.08
3.07
3.05
2.96
2.94
2.34
O
N
O
CH3
5c
1.91
1.03
2.00
4.10
1.00
0.99
2.02
1.02
3.16
10.0 9.5 9.0 8.5 8.0 7.5 7.0 6.5 6.0 5.5 5.0 4.5 4.0 3.5 3.0 2.5 2.0 1.5 1.0 0.5 0.0 -0.5
f1 (ppm)

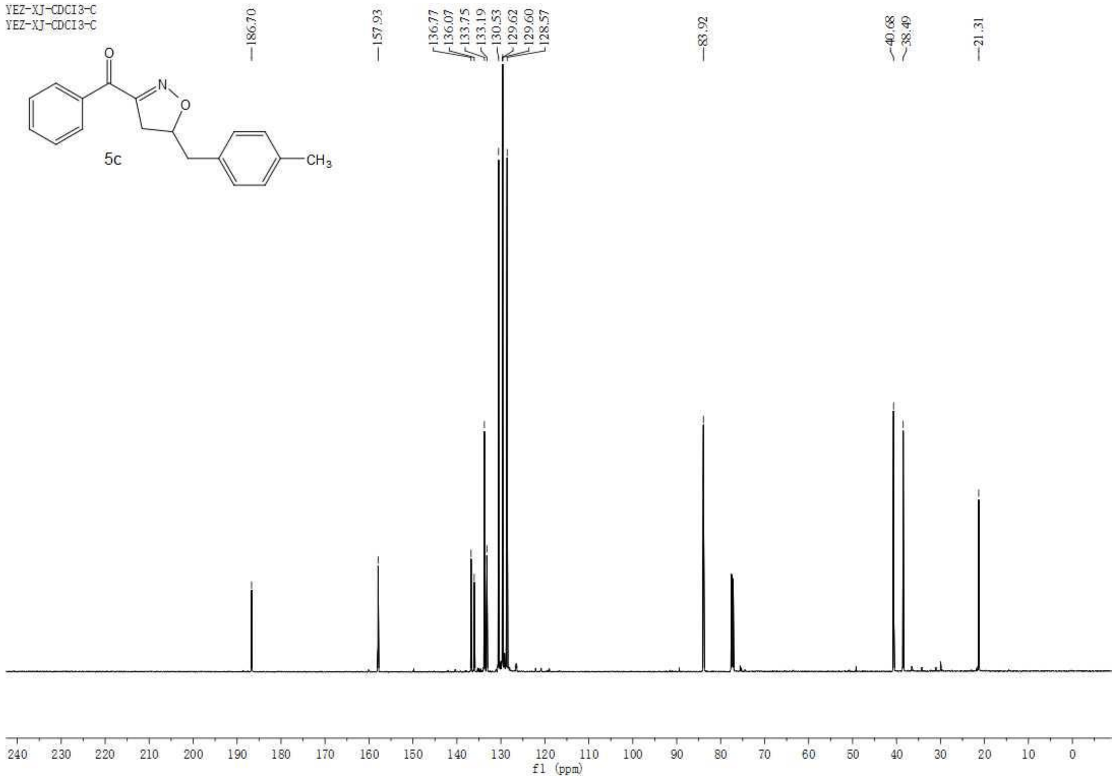
YEZ-XJ-CDCl3-C
YEZ-XJ-CDCl3-C
186.70
157.93
136.77
136.07
133.75
133.19
130.53
129.62
129.60
128.57
83.92
40.68
38.49
21.31
5c
CH3
240 230 220 210 200 190 180 170 160 150 140 130 120 110 100 90 80 70 60 50 40 30 20 10 0
f1 (ppm)

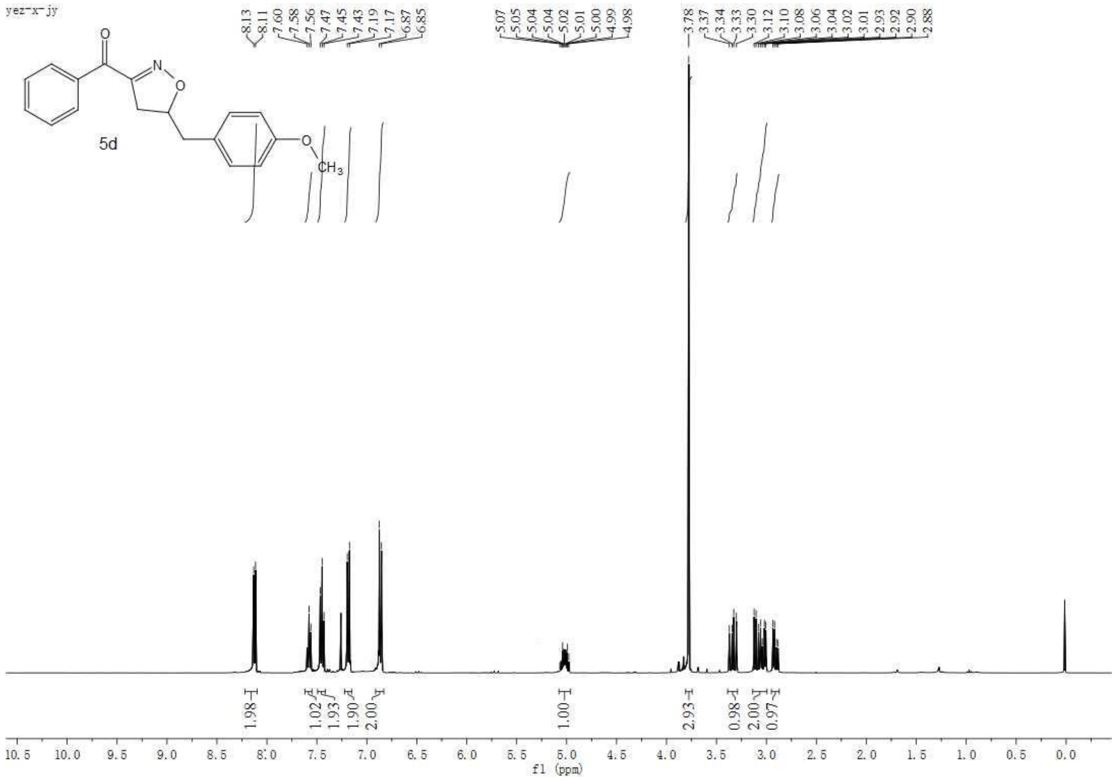
yez-x-jy
8.13
8.11
7.60
7.58
7.56
7.47
7.45
7.43
7.19
7.17
6.87
6.85
5.07
5.05
5.04
5.04
5.02
5.01
5.00
4.99
4.98
3.78
3.37
3.34
3.33
3.30
3.12
3.10
3.08
3.06
3.04
3.02
3.01
2.93
2.92
2.90
2.88
5d
1.98
1.02
1.93
1.90
2.00
1.00
2.93
0.98
2.00
0.97
10.5 10.0 9.5 9.0 8.5 8.0 7.5 7.0 6.5 6.0 5.5 5.0 4.5 4.0 3.5 3.0 2.5 2.0 1.5 1.0 0.5 0.0
f1 (ppm)

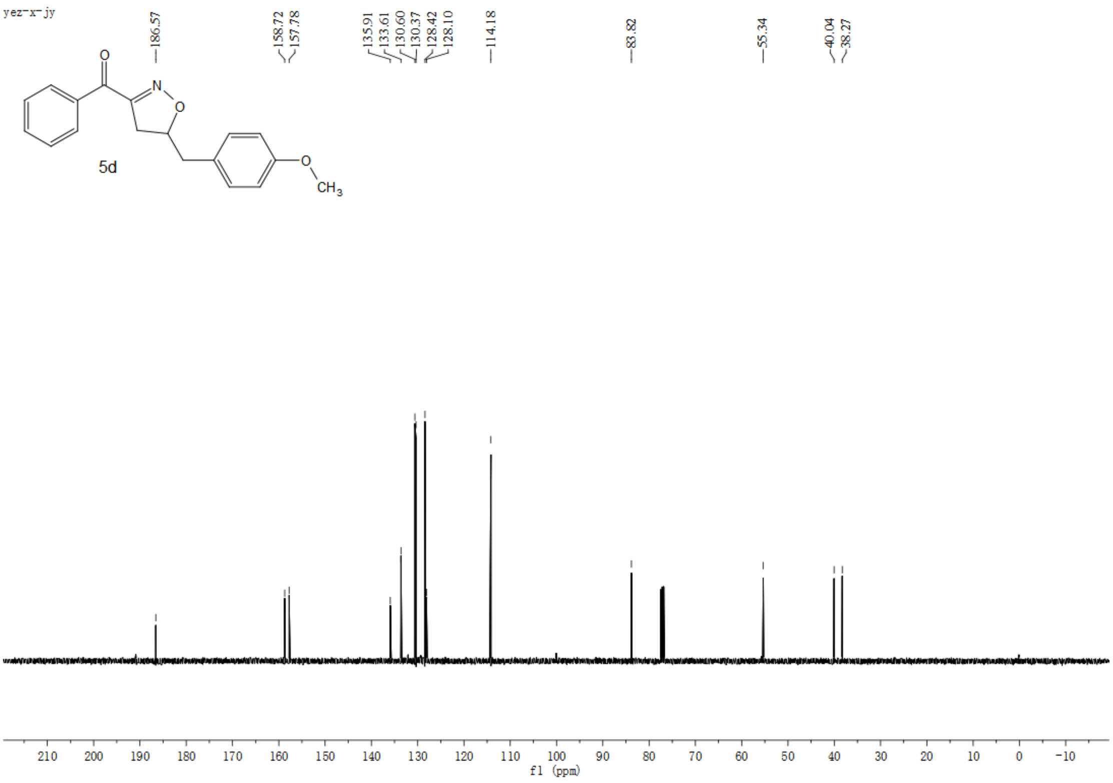
yez-x-jy
5d
186.57
158.72
157.78
135.91
133.61
130.60
130.37
128.42
128.10
114.18
83.82
55.34
40.04
38.27
f1 (ppm)

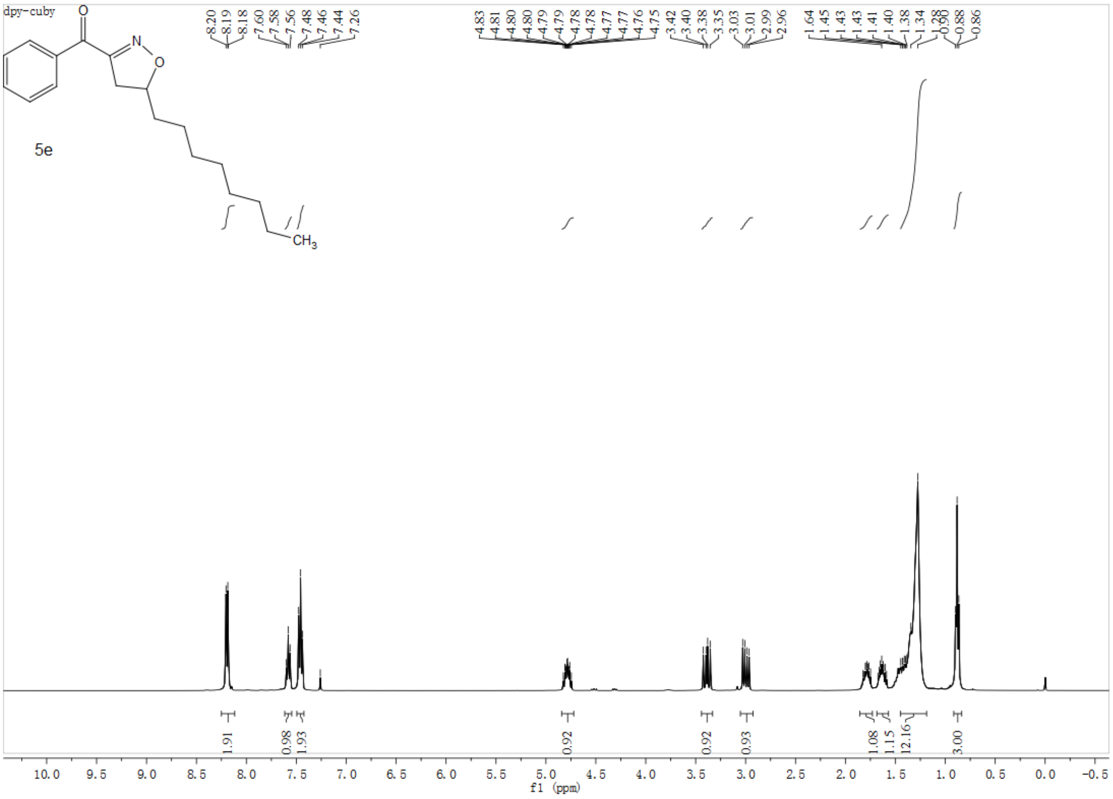
dpy-cuby
5e
8.20
8.19
8.18
7.60
7.58
7.56
7.48
7.46
7.44
7.26
4.83
4.81
4.80
4.80
4.79
4.79
4.78
4.78
4.77
4.77
4.76
4.75
3.42
3.40
3.38
3.35
3.03
3.01
2.99
2.96
1.64
1.45
1.43
1.43
1.41
1.40
1.38
1.34
1.28
0.90
0.88
0.86
1.91
0.98
1.93
0.92
0.92
0.93
1.08
1.15
12.16
3.00
f1 (ppm)

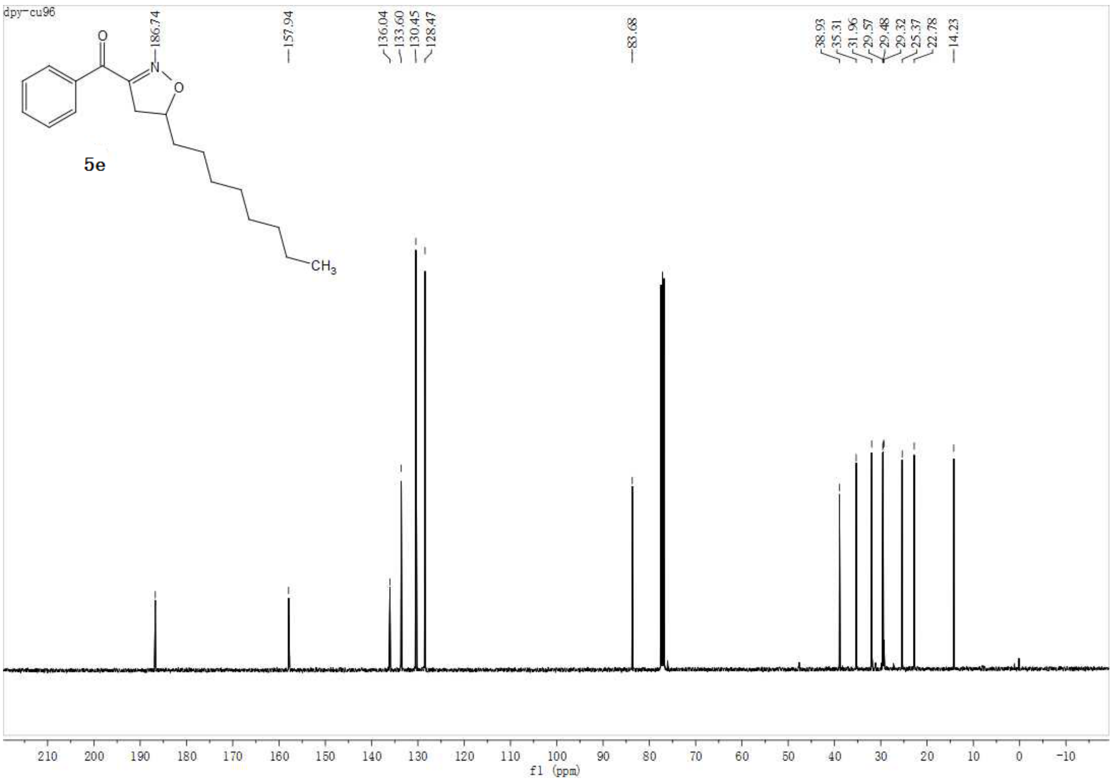
dpy-cu96
186.74
157.94
136.04
133.60
130.45
128.47
83.68
38.93
35.31
31.96
29.57
29.48
29.32
25.37
22.78
14.23
CH3
5e
f1 (ppm)

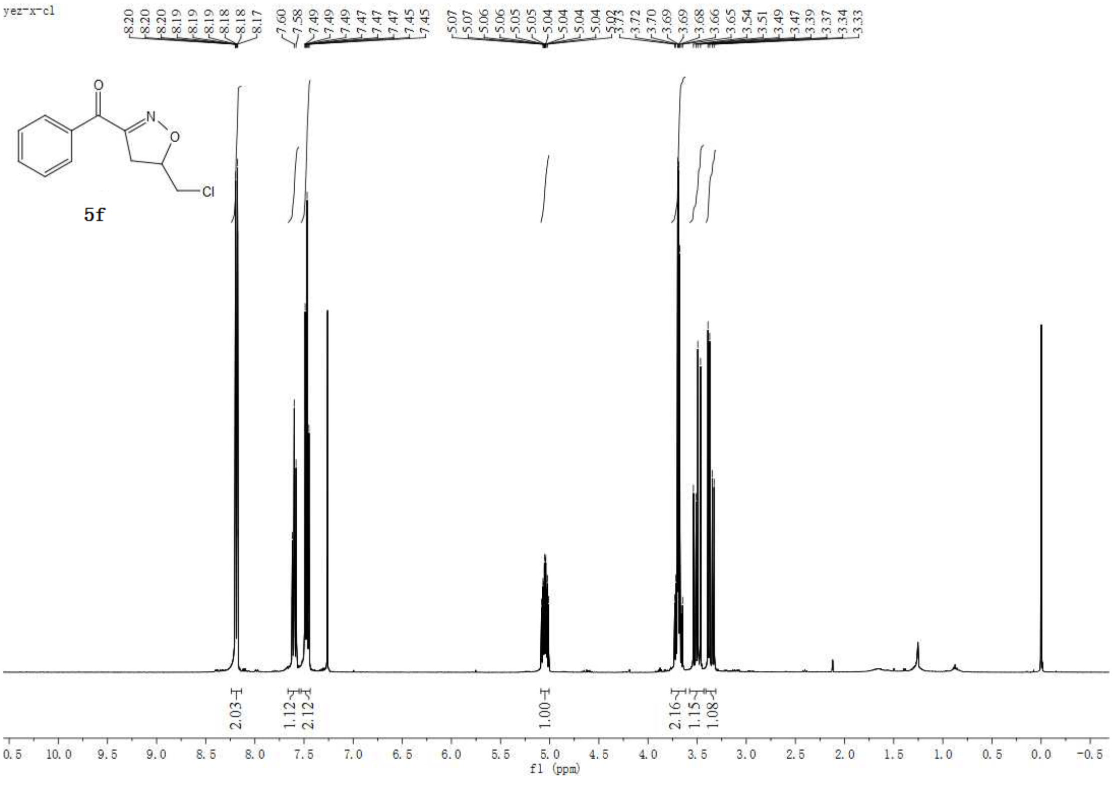
yez-x-cl
8.20
8.20
8.20
8.19
8.19
8.19
8.18
8.18
8.17
7.60
7.58
7.49
7.49
7.49
7.47
7.47
7.47
7.45
7.45
5.07
5.07
5.06
5.06
5.05
5.05
5.04
5.04
5.04
5.04
5.02
3.73
3.72
3.70
3.69
3.69
3.68
3.66
3.65
3.54
3.51
3.49
3.47
3.39
3.37
3.34
3.33
Cl
5f
2.03
1.12
2.12
1.00
2.16
1.15
1.08
f1 (ppm)

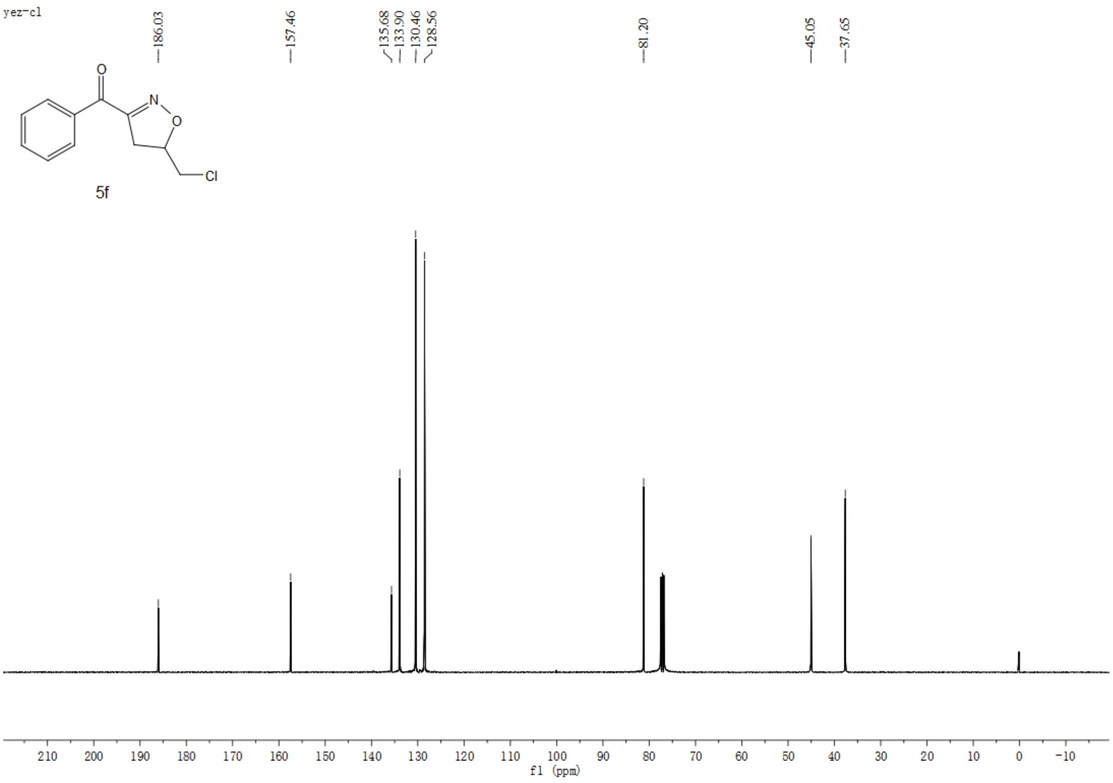
yez-cl
186.03
157.46
135.68
133.90
130.46
128.56
81.20
45.05
37.65
O
N
O
Cl
f1 (ppm)

5f

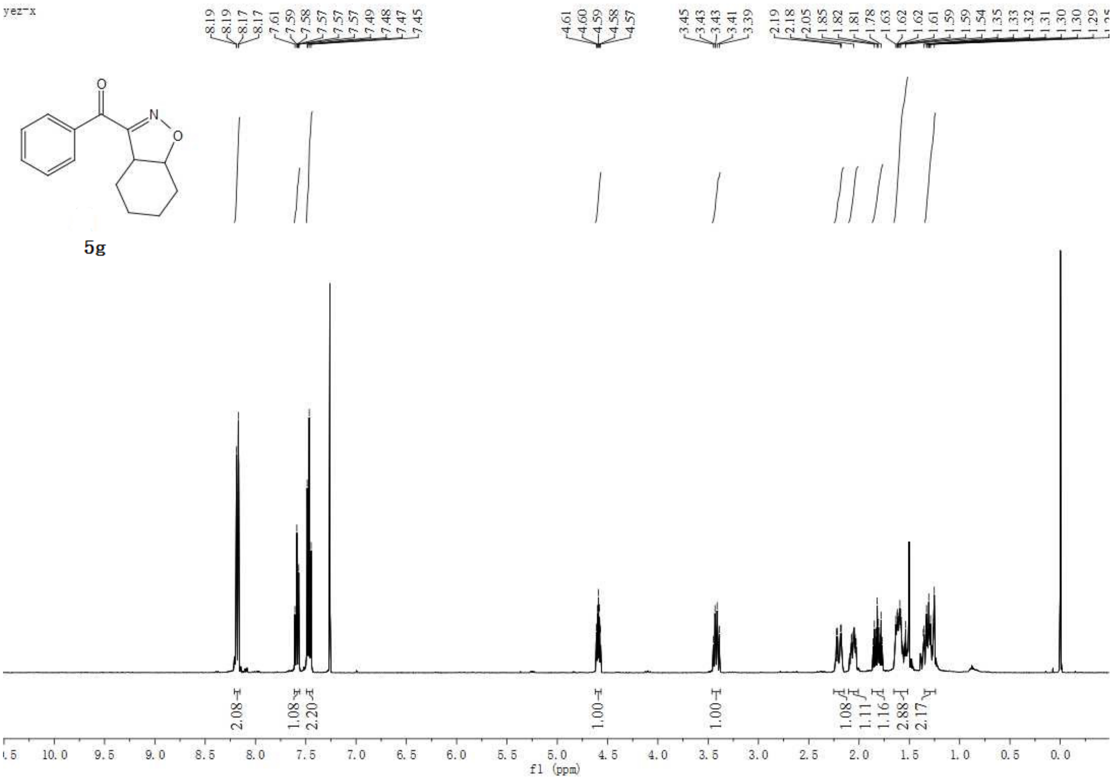
yez-x
8.19
8.19
8.17
8.17
7.61
7.59
7.58
7.57
7.57
7.57
7.49
7.48
7.47
7.45
4.61
4.60
4.59
4.58
4.57
3.45
3.43
3.43
3.41
3.39
2.19
2.18
2.05
1.85
1.82
1.81
1.78
1.63
1.62
1.62
1.61
1.59
1.59
1.54
1.35
1.33
1.32
1.31
1.30
1.30
1.29
O
N
O
2.08
1.08
2.20
1.00
1.00
1.08
1.11
1.16
2.88
2.17
f1 (ppm)

5g

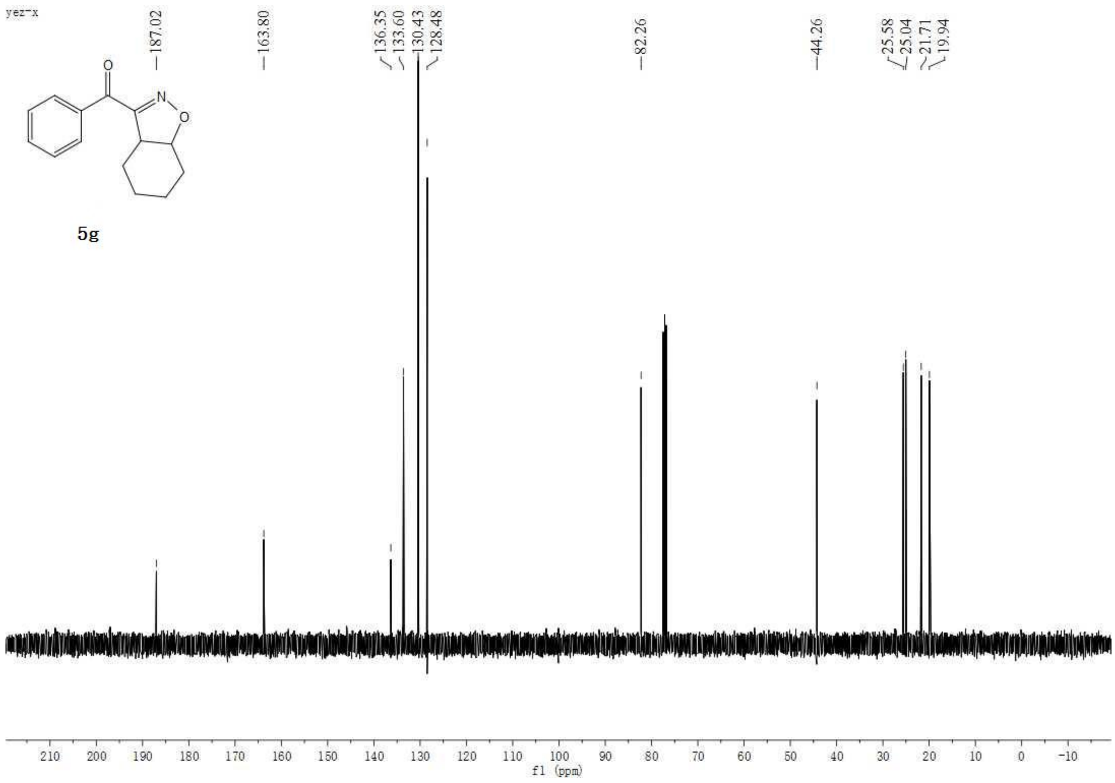
yez-x
187.02
163.80
136.35
133.60
130.43
128.48
82.26
44.26
25.58
25.04
21.71
19.94
5g
f1 (ppm)

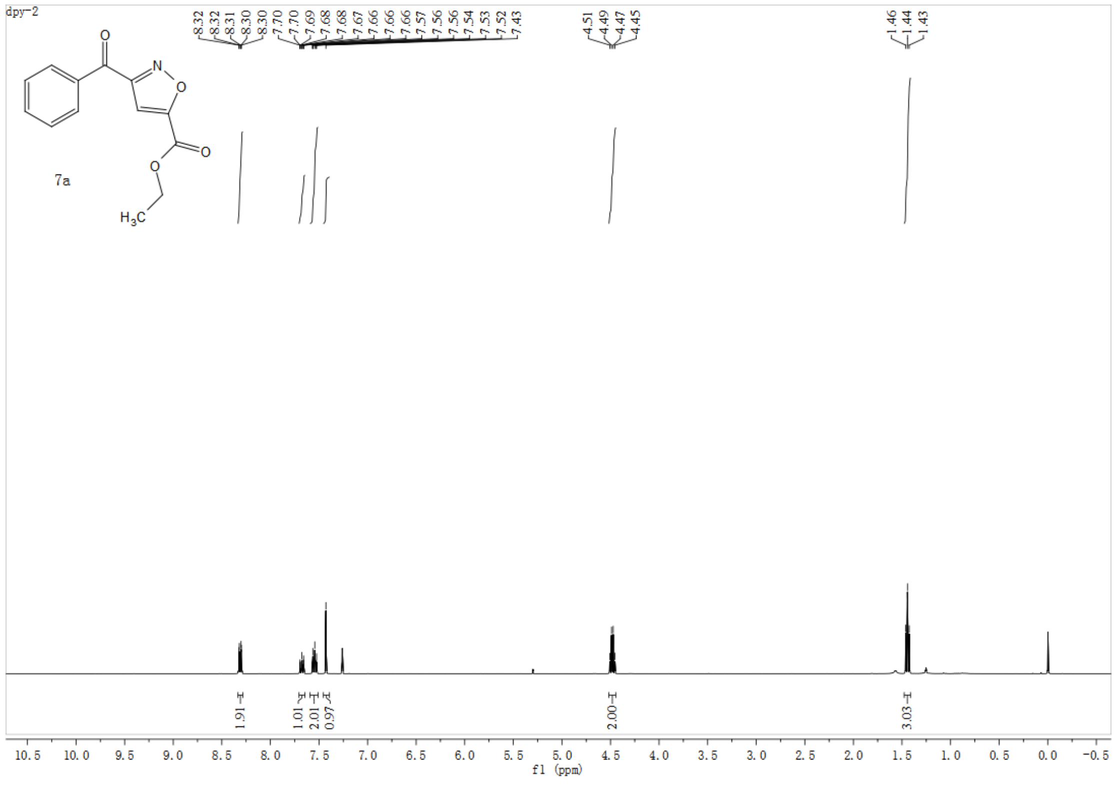
dpy-2
8.32
8.32
8.31
8.30
8.30
7.70
7.69
7.68
7.68
7.67
7.66
7.66
7.66
7.57
7.56
7.56
7.54
7.53
7.52
7.43
4.51
4.49
4.47
4.45
1.46
1.44
1.43
7a
1.91
1.01
2.01
0.97
2.00
3.03
f1 (ppm)

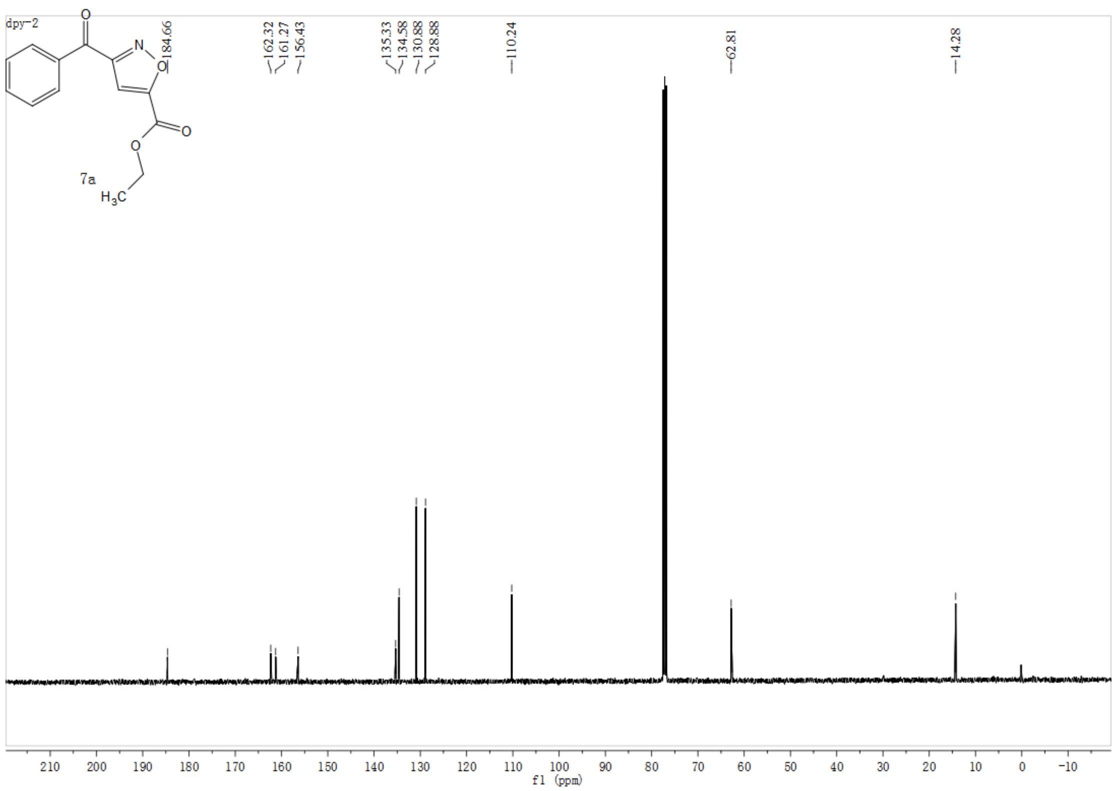
dpy-2
7a
184.66
162.32
161.27
156.43
135.33
134.58
130.88
128.88
110.24
62.81
14.28
f1 (ppm)

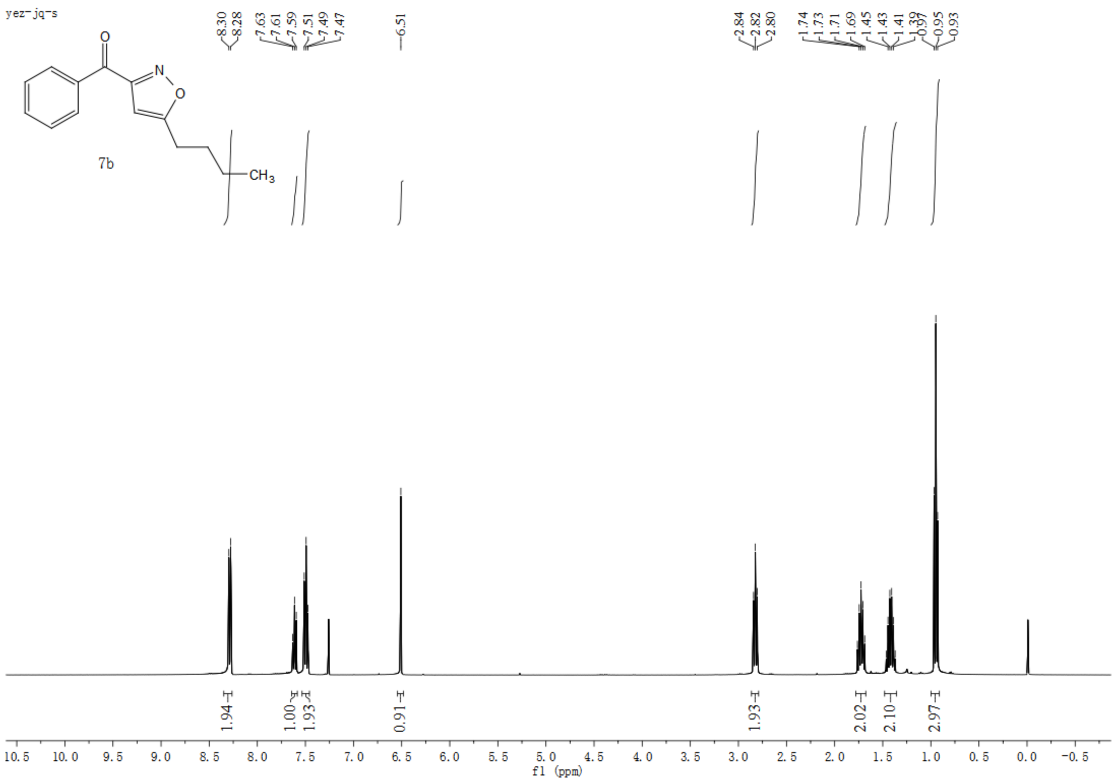
yez-jq-s
7b
8.30
8.28
7.63
7.61
7.59
7.51
7.49
7.47
6.51
2.84
2.82
2.80
1.74
1.73
1.71
1.69
1.45
1.43
1.41
1.39
0.97
0.95
0.93
1.94
1.00
1.93
0.91
1.93
2.02
2.10
2.97
f1 (ppm)

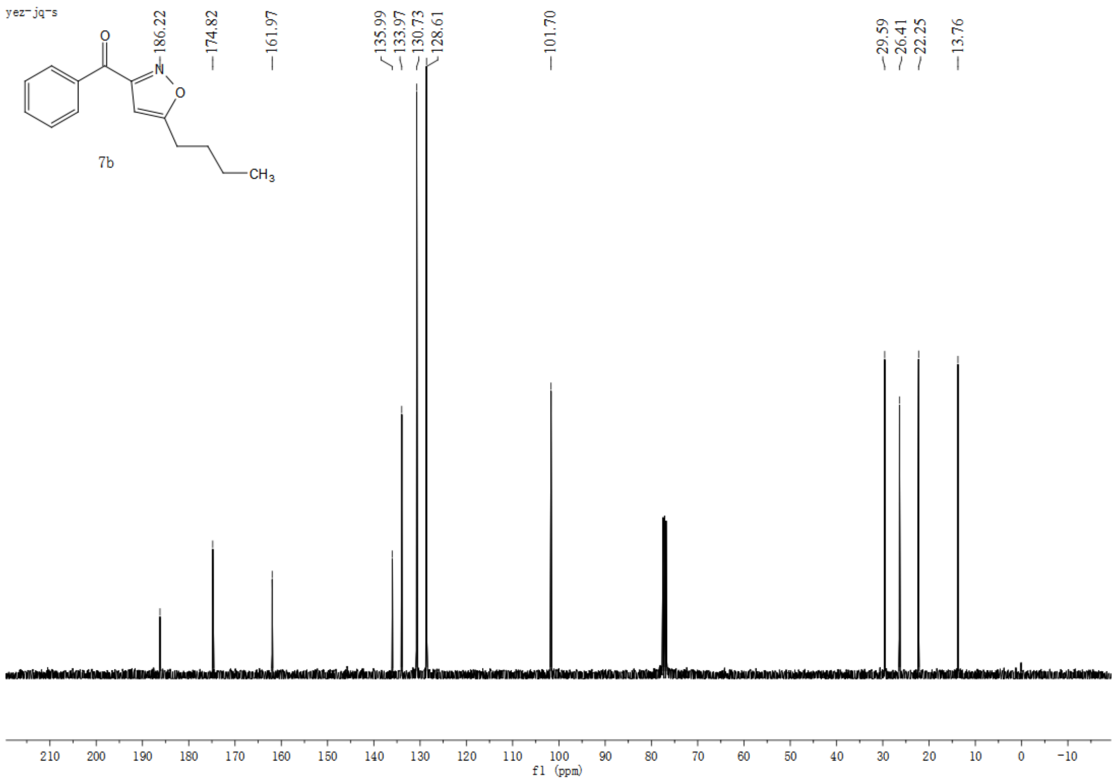
yez-jq-s
186.22
174.82
161.97
135.99
133.97
130.73
128.61
101.70
29.59
26.41
22.25
13.76
O
N
O
7b
CH3
210 200 190 180 170 160 150 140 130 120 110 100 90 80 70 60 50 40 30 20 10 0 -10
f1 (ppm)
